# Whole transcriptome sequencing reveals neutrophils’ transcriptional landscape associated with active tuberculosis

**DOI:** 10.3389/fimmu.2022.954221

**Published:** 2022-08-18

**Authors:** Xingzhu Geng, Xiaolin Wu, Qianting Yang, Henan Xin, Bin Zhang, Dakuan Wang, Liguo Liu, Song Liu, Qi Chen, Zisen Liu, Mingxia Zhang, Shouguo Pan, Xiaobing Zhang, Lei Gao, Qi Jin

**Affiliations:** ^1^ NHC Key Laboratory of Systems Biology of Pathogens, Institute of Pathogen Biology, and Center for Tuberculosis Research, Chinese Academy of Medical Sciences and Peking Union Medical College, Beijing, China; ^2^ Guangdong Key Laboratory for Emerging Infectious Diseases, Shenzhen Key Laboratory of Infection & Immunity, Shenzhen Third People’s Hospital, Shenzhen, China; ^3^ Center for Diseases Control and Prevention of Zhongmu, Zhengzhou, China

**Keywords:** active tuberculosis, RNA sequencing, neutrophil, interferon signaling, NF-κB signaling, biomarker

## Abstract

Neutrophils have been recognized to play an important role in the pathogenesis of tuberculosis in recent years. Interferon-induced blood transcriptional signatures in ATB are predominantly driven by neutrophils. In this study, we performed global RNA-seq on peripheral blood neutrophils from active tuberculosis patients (ATB, n=15); latent tuberculosis infections (LTBI, n=22); and healthy controls (HC, n=21). The results showed that greater perturbations of gene expression patterns happened in neutrophils from ATB individuals than HC or those with LTBI, and a total of 344 differentially expressed genes (DEGs) were observed. Functional enrichment analysis showed that besides the interferon signaling pathway, multiple pattern recognition receptor pathways were significantly activated in ATB, such as NOD-like receptors and Toll-like receptors. Meanwhile, we also observed that the expression of genes related to endocytosis, secretory granules, and neutrophils degranulation were downregulated. Our data also showed that the NF-κB signaling pathway might be inhibited in patients with ATB, which could increase *Mycobacterium tuberculosis* survival and lead to active tuberculosis status. Furthermore, we validated the accuracy of some differentially expressed genes in an independent cohort using quantitative PCR, and obtained three novel genes (*RBM3, CSRNP1, SRSF5*) with the ability to discriminate active tuberculosis from LTBI and HC.

## Introduction

Tuberculosis is one of the most serious infectious diseases endangering human health, and about a quarter of the world’s population is infected with *Mycobacterium tuberculosis* (1). Tuberculosis was the leading cause of death from a single infectious agent before the COVID-19 pandemic. Most people who are exposed to *M. tuberculosis* do not acquire active tuberculosis (ATB) or develop latent tuberculosis infection (LTBI), according to epidemiological statistics. Previous research findings have also revealed that many close contacts of TB patients have negative immunological tests (2), which may be because the early clearance of *M. tuberculosis* is mediated by an effective innate immune response (3). Besides alveolar macrophages and alveolar dendritic cells, neutrophils are one of the most abundant and one of the earliest immune cells recruited to the site of mycobacterial invasion (4, 5).

The role of neutrophils in the occurrence and development of tuberculosis is like a double-edged sword. It can not only play an anti-tuberculosis effect through innate immune responses but also interact with other immune cells to mediate specific immunity and play a role in regulating immunity (6). However, neutrophils may also lead to excessive inflammatory response and cause pathological damage (7). Furthermore, if neutrophils are unable to successfully destroy phagocytized *M. tuberculosis*, they can behave like “Trojan horses” for bacterial transmission (8, 9). Berry et al. previously reported an interferon-induced neutrophil-driven blood transcriptional signature in ATB by comparing ATB patients with healthy controls (HC), in their microarray analysis-based study (10). Recent studies have shown that neutrophils contribute to the severity of tuberculosis pathology, and their numbers decrease with completion of treatment (11). Intrinsic apoptotic pathway clearance of neutrophils is beneficial for *M. tuberculosis* infection control (12), and inhibition of neutrophil necrosis can be a strategy for host-targeted therapy (13).

Although the mechanism of tuberculosis immunity has not been entirely elucidated, the advancement and use of transcriptome sequencing technology is steadily altering our knowledge. Previous research using blood transcriptomes have advanced our knowledge of the host immune response mechanism and have aided in the differential diagnosis, treatment, and surveillance of TB (14, 15). However, recent investigations on tuberculosis transcriptomes have focused mostly on whole blood or peripheral blood mononuclear cell (PBMC) samples. Given the complexity of whole blood components, signal interference between diverse cell subsets may arise, and granulocytes (neutrophils, basophils, and eosinophils) were not included in PBMCs. Thus, whole-transcriptome sequencing of distinct cell subsets is required to investigate the variations in gene expression patterns of particular host cell types in response to *M. tuberculosis* infection states.

In this study, we performed an RNA-sequence analysis of CD15+ cells (primarily neutrophils) on our study subjects to provide a complete transcriptional landscape of the human peripheral blood neutrophils with different TB infection statuses. The data revealed changes in the transcriptional profile of ATB with significantly enhanced pathogen recognition and interferon-induced immune responses, while antibacterial functions such as phagocytosis, granule maturation, and neutrophil degranulation were inhibited. Interestingly, the NF-κB signaling pathway, which plays an important role in immunity and inflammation, was inhibited in ATB. Overall, this work could improve our understanding of the role of neutrophils in TB immunity and may provide clues for identifying transcriptional diagnostic biomarkers.

## Materials and methods

### Study design and participants

The overall study design and data analysis are summarized in **Supplementary Figure 1**. This investigation included 211 samples with three different infection statuses, including 58 in the RNA-seq cohort (15 patients with ATB, 22 patients with LTBI, and 21 HCs), and 143 in the qPCR cohort (31 patients with ATB, 53 patients with LTBI, and 59 HCs) (**Supplementary Table 1**). Active TB samples were collected from patients with active pulmonary tuberculosis at Shenzhen Third People’s Hospital, and all cases were confirmed by bacteriological examination (at least one positive result in sputum smear, *M. tuberculosis* culture, or nucleic acid amplification testing). All patients were originally diagnosed with ATB and were treated for <7 days with anti-tuberculosis medication. The LTBI and HC samples were based on participants from Xin’s study cohort in rural China (16). The LTBI group was defined by a positive interferon gamma release test (QuantiFERON^®^-TB Gold Plus), a lack of prior tuberculosis history, the absence of tuberculosis-related clinical symptoms, and an abnormal chest radiograph. The HC group was defined as having a negative interferon gamma release test, a lack of prior tuberculosis history, absence of tuberculosis-related clinical symptoms, and an abnormal chest X-ray. All participants were between the ages of 18 and 70 years, HIV-negative, not pregnant or lactating, not on immunosuppressive medicine, and did not have autoimmune illnesses. The Institute of Pathogen Biology of the Chinese Academy of Medical Sciences and the Shenzhen Third People’s Hospital Ethics Committee authorized this research, and all participants provided informed consent.

### Sample collection, library construction, and sequencing

We collected 5 mL peripheral blood from each participant and stored it in K2-EDTA anticoagulated blood collection tubes. Within 4 h, Dynabeads^®^ CD15 (Invitrogen) were used to extract CD15+ cells (mostly neutrophils and eosinophils). RNA was extracted and purified using the RNeasy Plus Mini Kit (QIAGEN) according to standard operating procedures. The SMARTer^®^ Stranded Total RNA-Seq Kit v2 (Takara) was used to construct RNA-seq libraries according to the manufacturer’s instructions. Sequencing was then performed using an Illumina NovaSeq6000 sequencer, and 150-bp paired-end reads were generated. RNA-Seq data for each sample are shown in **Supplementary Table 2**.

### Bioinformatics analysis

The raw data were quality-controlled and filtered by Trimmomatic software (17), and then aligned to the UCSC human reference genome (hg19) using STAR (18). HTSeq-count was used to generate read counts for individual transcripts in each sample (19), and mRNA abundance was normalized using Fragments Per Kilobase Million Reads (FPKM) values. Pairwise comparisons were performed using the DESeq2 algorithm to calculate the fold change in gene expression, and P-value and false discovery rate (FDR) were used for significance analysis (20). In this study, the screening criteria for differentially expressed genes (DEGs) were FDR<0.05 and the absolute value of log2FoldChange >1. Principal component analysis (PCA) was carried out with R package “factoextrap” (https://CRAN.R-project.org/package=factoextra). Based on the DEGs analysis, the R packages “ggplot2” (https://CRAN.R-project.org/web/packages/ggplot2) and “pheatmap” (http://CRAN.R-project.org/package= pheatmap) were used to draw volcano plots and heatmaps, respectively.

Functional enrichment analysis including Gene Ontology (GO), Kyoto Encyclopedia of Genes and Genomes (KEGG) pathway, Reactome pathway, WikiPathways, and Gene Set Enrichment Analysis (GSEA) were performed using the R package “ClusterProfile” (21, 22). A threshold of P-values <0.05 was set up as a cut-off for significant enrichment.

Protein–protein interaction (PPI) network and transcription factor (TF) target interaction networks were performed using a web-based tool of Metascape (https://metascape.org) (23). The protein interaction network was generated by STRING (24) and the Molecular Complex Detection (MCODE) algorithm (25) was applied to identify densely connected network components. The TF-target interaction networks were determined by a literature-curated TF–target interactions database TRRUST (26).

Weighted Gene Co-expression Network Analysis (WGCNA) was performed *via* an online web server ImageGP (http://www.ehbio.com/ImageGP/) using the top 3000 genes based on mean absolute deviation (MAD). Traversing all the given soft threshold power (maximum=30), when the scale-free topology index was >0.85, the minimum soft threshold power was obtained. Then, the modules were merged by setting the cut height value to 0.2, the depth split to 2, and the minimum module size to 25. Then, the correlation of gene modules and phenotypes was calculated and modules that were significantly related with distinct TB infection statuses were detected.

### RT-qPCR

We applied RT-qPCR to assess gene expression levels. Assays were performed on an ABI 7500 Fast Real-Time PCR System according to the manufacturer’s protocol using TaqMan™ Fast Advanced Master Mix (Thermofisher) and TaqMan™ Gene Expression Assay (Thermofisher) under standard conditions (**Supplementary Table 3**). The stably expressed *MYO1F* assessed by our laboratory was used to normalize the target gene, and the relative expression was calculated using the 2 ^-(ΔΔCt)^ method. Statistical analysis was performed using Kruskal–Wallis with Dunn’s multiple comparisons tests. We used the R package “pROC” to perform receiver operating characteristic (ROC) analysis and plot the ROC curve. A logistic regression model was constructed using the R package “glmnetc” for multigene combined ROC analysis.

## Results

### The gene expression pattern of the ATB group was significantly different from that of the LTBI and HC groups

**Figure 1:**
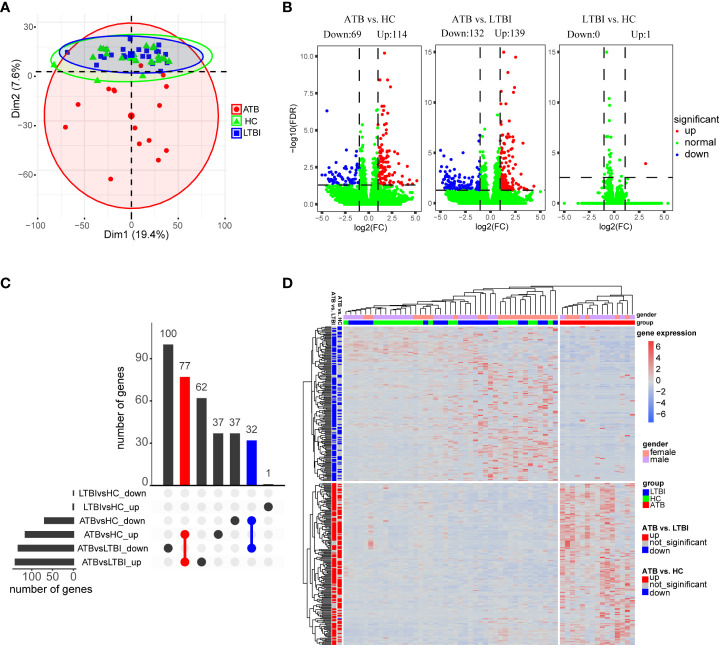
Analysis of differential expression of neutrophils in three groups of samples. **(A)** Principal component analysis of gene expression profiles grouped by different tuberculosis infection status. **(B)** Volcano plot shows the distribution of differentially expressed genes in pairwise comparisons among the three groups of samples. **(C)** Upset plot shows the overlap of the three sets of differentially expressed genes. **(D)** Hierarchical clustering heatmap of differentially expressed genes in all samples. Each row represents a gene, columns represent samples, and relative levels of gene expression are represented by color scale, with red for high expression and blue for low expression.

We used PCA to perform a preliminary assessment of the gene expression profiles of the three groups of samples. After normalizing the raw expression levels of each gene with DESeq2 for all samples, the top 5000 highly variable genes were extracted for PCA. As shown in **Figure 1A**, ATB showed significant differences compared with the LTBI and HC groups. However, the expression patterns did not show a clear separation between the LTBI and HC groups.

In total, as depicted in the volcano maps, we identified 183 DEGs in the ATB group versus the HC group (including 114 up-regulated genes and 69 down-regulated genes) and 271 DEGs in the ATB group versus the LTBI group (including 139 up-regulated genes and 132 down-regulated genes); only one gene was found to be significantly upregulated in the LTBI group compared to HCs (LTBI vs. HC) (**Figure 1B**, **Supplementary Table 4**). An Upset plot showed that 110 genes overlapped between ATB vs. HC and ATB vs. LTBI, and the direction of regulation (up- or down-regulation) was consistent (**Figure 1C**).

The DEGs of the ATB vs. HC and ATB vs. LTBI were shown in heatmap by hierarchical clustering, and the ATB group was clearly distinguishable from the other two groups (**Figure 1D**). Interestingly, both in the LTBI and HC groups, some of the ATB-downregulated genes were slightly higher in female than in male individuals, while no gender bias was observed in the ATB-upregulated genes.

### Transcriptomic comparison between ATB vs. HC and ATB vs. LTBI showed enrichment of similar functional pathways

To better understand the functions of the detected DEGs, we performed functional pathway enrichment analysis; additionally, to gain a more complete picture of the changes across the transcriptome and to explore whether genes with insignificant differential expression are also biologically significant, we also performed GSEA analysis. The results showed that most of the pathways enriched in ATB vs. HC and ATB vs. LTBI were similar, although the former was usually more significant.

**Figure 2:**
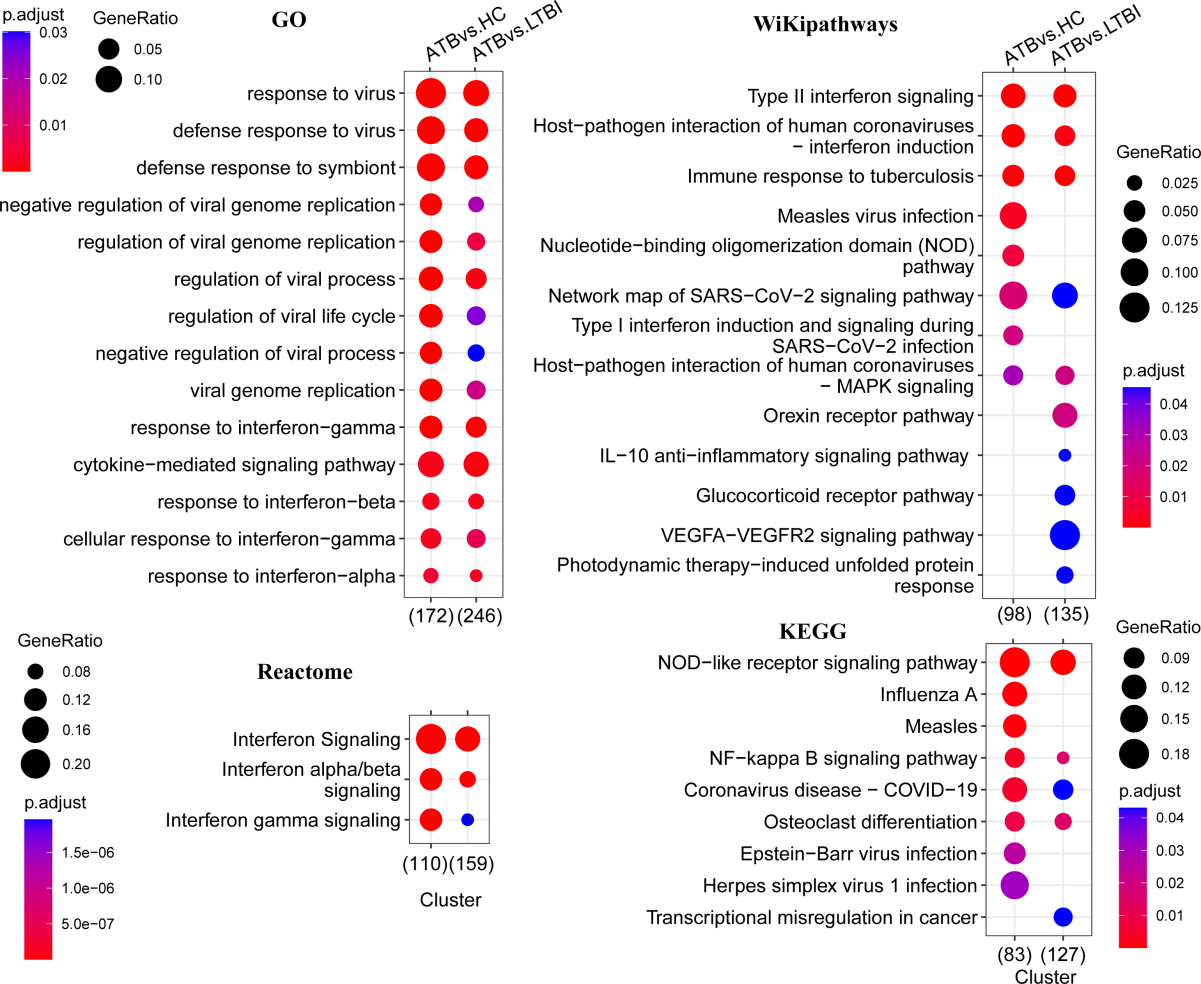
Functional enrichment analysis of differentially expressed genes. This figure shows the pathways that are significantly enriched in the differentially expressed genes (p < 0.05), and the GO enrichment analysis only shows the top 15. The color of the bubbles indicates significance (adjusted P-value), and the size corresponds to the proportion of genes with corresponding annotation.

GO analysis results showed that these DEGs were significantly enriched only in the biological process (BP) category. As shown in **Figure 2**, the top enriched terms of each comparison group mainly focused on the defense response to virus, the biological process involved in symbiotic interaction and cytokine-mediated signaling pathway. Other pathway analysis results showed significantly enriched NOD-like receptor signaling pathway, NF-kappa B signaling pathway, type I/II interferon signaling, and pathways in response to infection by multiple pathogens, including tuberculosis (**Figure 2**). Many guanylate-binding protein genes and interferon-inducible protein genes related to antiviral response, such as *GBP5, GBP4, IFI44, IFITM3*, and *IFIT3* were significantly increased in ATB patients (**Supplementary Figure 2**).

The significant pathways obtained by GSEA analysis were mostly consistent with the enrichment analysis of DEGs. In addition to these significant pathways enriched in DEGs, we also enriched the response to lipopolysaccharide, chemokine signaling pathway, nucleic acid metabolism, and toll-like receptor pathway (**Supplementary Figure 3**). Apoptosis and TNF-signaling pathways that were only significantly enriched in ATB vs. LTBI in the DEGs enrichment analysis were significantly enriched in both comparison groups in GSEA.

Differentially expressed genes were used to construct a protein interaction network based on the STRING database, and eight densely connected network components were identified from the constructed PPI network using the MCODE algorithm. The top four core subnetworks were mainly involved in interferon gamma signaling, interferon alpha/beta signaling, purine metabolism, and mRNA splicing (**Supplementary Figure 4**). Notably, the subnetwork associated with mRNA splicing was only observed in the DEGs of ATB vs. LTBI.

### NF-κB pathway was inhibited in active tuberculosis

**Figure 3:**
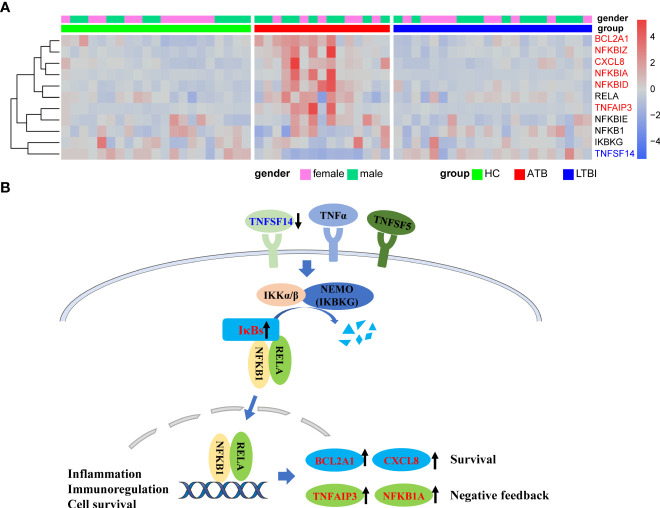
Upregulation of NFKB inhibitors in ATB might lead to inhibition of NFKB pathway activation. **(A)** Heat map of expression levels of some of the genes involved in the NFKB pathway. **(B)** Simplified schematic diagram of the canonical NFKB pathway. Downregulation of TNFSF14 results in insufficient activation of pathway initiation, and the overexpression of IκBs might inhibit nuclear translocation of NFkB dimers. Upregulation of the NFkB negative feedback regulator TNFAIP3 and NFKB1A further inhibited the pathway activation. Red font represents significantly upregulated genes in ATB, and blue font represents significantly downregulated genes in ATB.

The results of KEGG analysis showed that the NF-κB signaling pathway was significantly enriched, and multiple genes involved in the canonical activation pathway were significantly differentially expressed in the ATB group (**Figure 3**). The expression of *TNFSF14*, one of the upstream activators of the NF-κB pathway, was significantly downregulated, which may lead to insufficient initial activation of the NF-κB pathway. Genes encoding NF-kappa B inhibitors (IκBs) such as *NFKBIA, NFKBID* and *NFKBIZ* were significantly upregulated. IκBs can bind to NF-κB (p50/p65) and restrict the translocation of the NF-κB complex to the nucleus for transcriptional regulation. The downstream negative feedback regulators *TNFAIP3* and *NFKB1A* were also upregulated, which may further inhibit the activation of NF-κB signaling pathway. However, no significant changes were observed both in *IKBKG*, which positively regulates the NF-κB pathway, and *NFKB1*, *RELA*, which encodes NF-κB (p50/p65) dimers. In addition, the expression levels of BCL2A1 and CXCL8, which are related to cell survival, were significantly upregulated.

### Genes related to mRNA splicing were downregulated in latent tuberculosis infections

**Figure 4:**
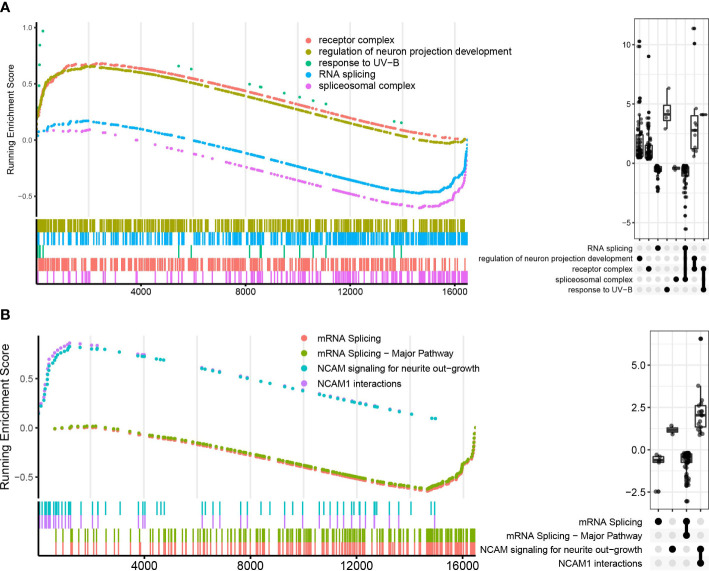
GO and Reactome pathways enrichment analysis in LTBI vs. HC in GSEA enrichment plot. **(A)** shows the top five pathways in GO enrichment analysis, **(B)** shows the top four pathways in Reactome enrichment analysis. The upper curves represent the running sum of enrichment scores, the bottom part of the figure shows the position of genes that are related to certain pathways. The Upset plot on the right visualizes the metric distribution of genes belonging to a pathway or multiple pathways.

Because the LTBI vs. HC group did not undergo enough screening for DEGs for enrichment analysis, GSEA analysis could complement it. In GO, KEGG, and Reactome enrichment analysis, it was found that RNA splicing-related pathways were significantly down-regulated in the LTBI group compared with the HC (**Figure 4**, **Supplementary Figure 3**). This echoes that the fourth sub-network in the PPI network analysis of DEGs contains only genes for ATB vs. LTBI.

### Modular analysis of transcriptional profiles in different tuberculosis infection states

**Figure 5:**
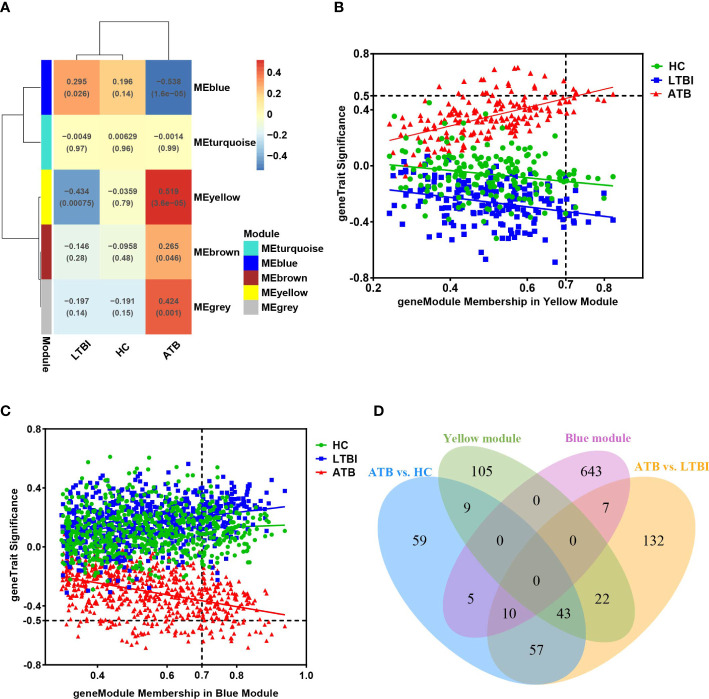
**(A)** The module-traits correlation matrix heat map of WGCNA, wherein each row represents a module eigengene and each column represents different clinical group. The color gradient indicates the strength of the correlation (blue represents negative correlation, red represents positive correlation), and the numerical value in each cell indicates the Pearson’s correlation value and P-value (in brackets). **(B, C)** represent the relationship graphs of Module membership and Gene significance of the yellow and blue modules, respectively. Genes with Module membership >0.7 and |Gene significance| >0.5 are hub genes in the module. **(D)** Venn diagram showing overlap of key modules’ genes and differentially expressed genes.

We used WGCNA analysis to decipher the gene co-expression relationship between host neutrophils with distinct TB infection statuses. We identified and clustered four distinct co-expression modules (**Supplementary Figure 5**). The result shows that the yellow module was positively correlated with ATB (Pearson’s correlation value: 0.519, P=3.6e-05), whereas the blue module was negatively correlated with ATB (Pearson’s correlation value: -0.538, P=1.6e-05) (**Figure 5A**). As a result, we evaluated the correlation between genes and traits (gene significance) as well as the correlation between modules and genes (module membership) in the yellow and blue modules, respectively, and plotted the results using scatter plots (**Figure 5B, C**). Additionally, these two module genes expression heatmaps enabled us to clearly identify ATB from LTBI and HC (**Supplementary Figures 6A, B**).

We identified 180 and 665 genes in the yellow and blue key modules, respectively (**Supplementary Table 5**). The yellow module’s genes had a greater number of overlaps with DEGs (74 genes), while the blue module’s genes had just 22 overlaps (**Figure 5D**). To better understand the functions of these genes, we performed functional enrichment analysis. The findings of the yellow module genes enrichment results were comparable to those of the previous DEGs enrichment analysis and GSEA (**Supplementary Figure 7**). These genes were predominantly associated with defense responses to virus, cytokine-mediated signaling pathway, and interferon signaling, NOD-like receptor signaling pathway and NF-κB-related signaling pathway.

**Figure 6:**
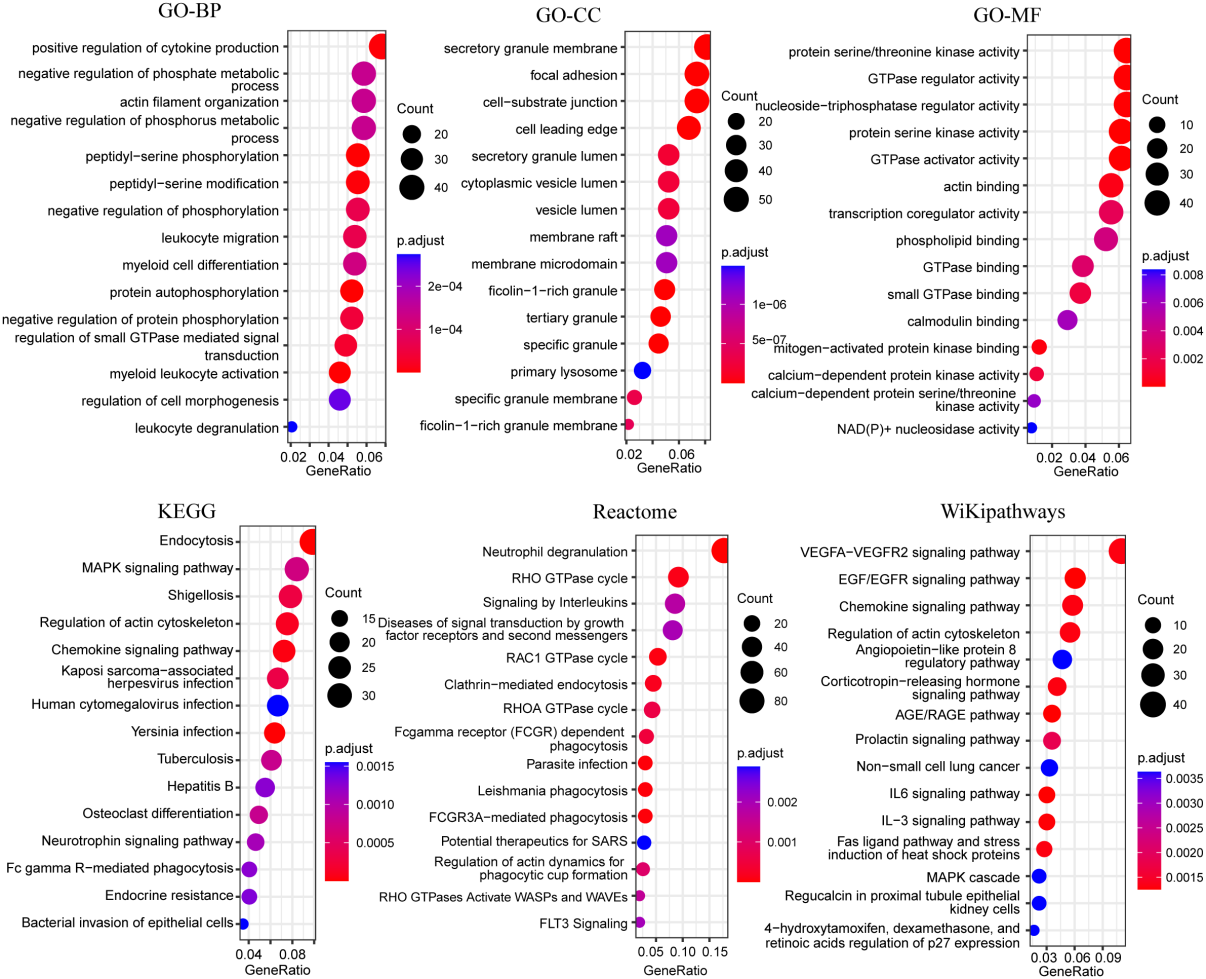
Bubble plots of functional enrichment analysis of genes in blue modules, showing only the top 15 significantly enriched pathways. The color of the bubbles indicates significance (adjusted P-value), and the size corresponds to the count of genes with corresponding annotation.

Unlike most of the previous up-regulated genes, the blue module genes were significantly down-regulated in the ATB group. The results of the GO enrichment analysis indicated that these genes were primarily enriched in cytokine production, phosphate metabolism, myeloid leukocyte activation, regulation of protease kinases, protein phosphorylation, neutrophil-secreted granules, and regulation of GTPase activity (**Figure 6**). Endocytosis, MAPK signaling pathway, neutrophil degranulation, RHO GTPase cycle, phagocytosis, and VEGFA-VEGFR2 signaling pathway were also significantly enriched in other pathways’ enrichment analysis (**Figure 6**). Downregulation of these pathways’ related genes might lead to diminished antibacterial function of neutrophils.

### Validation of DEGs and signature genes for distinguishing ATB in an independent cohort

Nine target genes were selected and validated using qPCR, with preliminary validation in 76 validation populations (including 24 in the ATB group, 24 in the LTBI group, and 28 in the HC group). Statistical analysis found that the expression of *GBP5, SRSF5, CSRNP1, RBM3*, and *CCNL1* genes were significantly increased in the ATB group. There was no statistical difference in the *PIM2* gene, but the overall dispersion was relatively high, and the trend was biased towards an increase. The negative control genes *SHKBP1* and *ITM2B* were not statistically different among the three groups. The only significant DEG *HBB* screened in LTBI vs. HC was verified to have no significant difference. The *HBB* gene, which encodes the β subunit of hemoglobin, was shown to have very low expression in neutrophils by qPCR, which may be the cause of the inconsistency. **Supplementary Figure 8** shows the relative expression levels of different groups of target genes.

**Figure 7:**
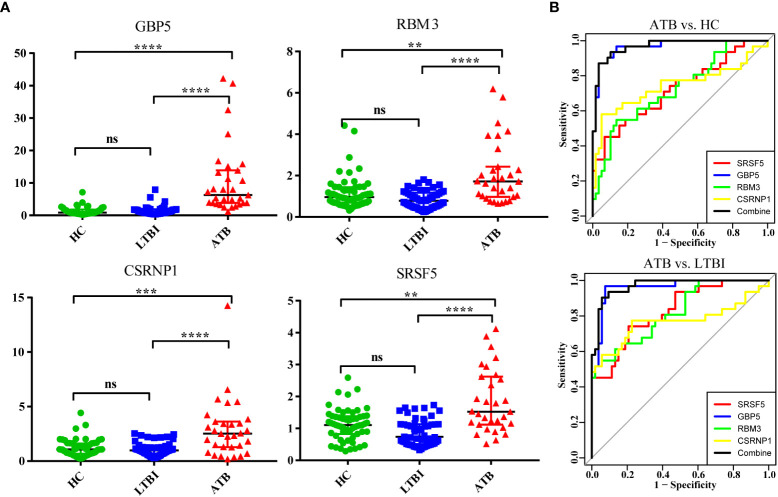
Validation of four significantly differentially expressed genes in an independent cohort by qPCR. **(A)** The scatter plot shows the relative expression of each gene in all samples. **(B)** The ROC curve used ATB as the case group, and LTBI and HC as the control group, respectively. ns (not significant) P > 0.1234, *P < 0.0332, **P < 0.0021, ***P < 0.0002, and ****P < 0.0001.

We selected four genes (*GBP5, SRSF5, CSRNP1, RBM3*) with the most significant differences to expand the sample size for further verification and ROC analysis (**Figure 7**). A total of 143 samples were included in the analysis (31 in the ATB group, 53 in the LTBI group, and 59 in the HC group). It is obvious that the expression levels of these four genes were significantly increased in the ATB group. Except for the *SRSF5* gene expression being slightly lower in the LTBI group than in the HC group, there was no significant difference in the expression levels of other genes between these two groups. We used the ATB group as the case group, and the LTBI and HC groups as the control group for ROC analysis. The results showed that the *GBP5* gene had the best discriminative ability (AUC>0.9), and the other three newly discovered genes also had potential diagnostic value with AUC>0.7. The combined use of 4 genes performed slightly better than GBP5 alone.

## Discussion

Whether it is the innate immune response during the early stages of tuberculosis infection, the regulation of adaptive immunity during infection, or the pathological damage to the body following disease onset, the complexity of the role of neutrophils in the process of tuberculosis infection is self-evident. In this study, we applied transcriptome sequencing to investigate peripheral blood neutrophils from HCs and patients with ATB and LTBI to achieve a better understanding of the gene expression patterns of host neutrophils in different *M. tuberculosis* infection stages. From the results of PCA and DEG analyses, the gene expression patterns of peripheral blood neutrophils from *M. tuberculosis* LTBI patients and HCs were comparable, but those from ATB patients exhibited more gene disturbance. This echoed their clinical presentation.

As professional phagocytes, the recognition and phagocytosis of invading *M. tuberculosis* is an important mechanism of neutrophil anti-TB immunity. Numerous pattern recognition receptors are expressed on the surface of neutrophils for pathogen recognition that trigger a series of downstream cellular events (27). In this study, we observed the enrichment of multiple pattern recognition receptor pathways such as NLRs, TLRs, and CLRs, which further confirmed the enhanced recognition ability of neutrophils after *M. tuberculosis* infection. NOD-like receptors are pattern-recognition receptors located in the cytoplasm that recognize pathogen components and activate inflammatory pathways (28). Toll-like receptors are pattern-recognition receptors on the cell surface that can recognize the mycobacterial cell wall components and play an important role in the initiation and coordination of host anti-TB immune responses in the early stage of infection. Through GSEA analysis, we observed some Toll-like receptor signaling pathways. These receptors recruit a variety of adaptor molecules such as MyD88 and TRAF6, thereby activating a variety of downstream signaling pathways, such as NF-κB and MAPK (29, 30). In addition, the phagocytosis of mycobacteria by neutrophils was also mediated by opsonized receptors, and the expression of Fc receptor-encoding genes *FCGR1A* and *FCGR1B* was significantly upregulated in the ATB group. However, high expression of these pattern-recognition receptors and opsonization receptors does not imply that neutrophils can completely phagocytose and kill *M. tuberculosis*. As a successful pathogen, *M. tuberculosis* has developed multiple mechanistic strategies to escape elimination by the immune system. We observed significant enrichment of endocytosis and phagocytosis in the downregulated module, which may imply a weakening of related functions.

Granules are a special storage organelle evolved by neutrophils to store a large amount of antibacterial substances produced by them. Phagosomes containing *M. tuberculosis* fuse with granules during maturation. Neutrophils recognize the invasion of pathogens through immune receptors and initiate degranulation, releasing their contents to kill pathogens, but excessive degranulation can also lead to tissue damage. Among the gene modules significantly downregulated in the ATB group, pathways such as secretory granule, protein kinase activation, and neutrophil degranulation were significantly enriched, indicating that related functions may be inhibited. Studies have shown that *Yersinia pestis* can inhibit neutrophil degranulation, thereby contributing to the survival of *Y. pestis* in primary pneumonic plague (31). Similarly, Y. pseudotuberculosis impairs the antibacterial capacity of neutrophils by inhibiting neutrophil phagocytosis and degranulation (32).

Different fates of neutrophils infected with *M. tuberculosis* also lead to different infection outcomes. Neutrophil necrosis facilitates the survival of *M. tuberculosis*, while phagocytosis by phagocytes after apoptosis helps to control *M. tuberculosis* infection (33, 34). Apoptotic neutrophils are phagocytosed by macrophages through the process of endocytosis, which can not only remove neutrophils and prevent excessive inflammation, but also use neutrophil granule proteins for antibacterial defense and change the production of cytokines (35). However, virulent mycobacteria have multiple mechanisms to inhibit apoptosis to escape immune killing. There is evidence that the ESAT-6 protein secreted by *M. tuberculosis* can induce neutrophil necrosis (36). We found that in the ATB group, many genes that promote apoptosis such as *CASP5, DDIT3, G0S2, PMAIP1*, and *XAF1* were significantly upregulated, but some genes that were beneficial to cell survival (*BCL2A1, DNAJB9, SELENOK*) were also upregulated. Perhaps there is a contest between the host’s anti-TB immune response and the immune escape of *M. tuberculosis*.

Transcriptional regulation is essential for cells to respond to environmental perturbations, and the simplest and most direct approach of transcriptional regulation is direct regulation of target gene expression through transcription factors. We compared the observed DEGs to the transcriptional regulatory networks in the TRRUST database and discovered that these DEGs were mostly regulated by *NFKB1, RELA, STAT3, STAT1*, and *ATF4* (**Supplementary Figure 9**).

Nuclear factor Kappa B is a key regulator of immune and inflammatory responses, and the canonical NF-κB pathway can be activated by various pathogen-associated molecular patterns (PAMPs) or stimulatory signals such as inflammatory cytokines (37, 38). The heterodimeric RELA-NFKB1 (p50-p65) complex is the most abundant form of the canonical NF-kappa-B activation pathway (39). Our results showed that the gene expression levels of multiple inhibitors of NF-κB (*NFKBIA, NFKBID, NFKBIZ*) were significantly up-regulated in the ATB group. In resting cells, NF-κB forms a complex with IκBs and is sequestered in the cytoplasm in an inactive form, limiting the activation of the NF-κB signaling pathway. When IκBs are modified and degraded, NF-κB translocates to the nucleus and regulates the transcription of a large number of genes, including antimicrobial peptides, cytokines, immunoregulatory proteins, and anti-apoptotic proteins (40). In addition, the downstream negative feedback regulator *TNFAIP3* was upregulated, which may further inhibit the activation of the NF-κB signaling pathway. Previous studies have shown that, in macrophages, *M. tuberculosis* inhibits NF-κB activation by down-regulating miRNA Let-7f, through attenuating the inhibition of A20 (*TNFAIP3*) by Let-7f (41). Therefore, the high expression of *TNFAIP3* in neutrophils may also be regulated by *M. tuberculosis*. We speculate that a similar mechanism may also exist in neutrophils. Therefore, *M. tuberculosis* may inhibit the activation of NF-κB by manipulating neutrophils, thereby attenuating inflammatory signaling and apoptosis, which is beneficial to the survival of *M. tuberculosis*.

The Jak–STAT pathway is thought to be the immune system’s central communication node (42), and mice lacking the *STAT1* gene died from severe infection, suggesting that this gene is vital in pathogen response (43). Berry et al. have shown that interferon-induced activation of the JAK1/2-STAT1 pathway occurs in the peripheral blood cells of individuals with active TB, especially neutrophils (10). *STAT1* and *STAT3* belong to a family of signal transducer and transcription activators that play critical regulatory functions in cell cycle, cell survival, and immunological responses. They may be activated by common cytokines and growth factors but play essentially antagonistic roles (44). NSP1 and ORF6 from SARS-CoV-2 may impair *STAT1* function in COVID-19 patients, resulting in compensatory *STAT3* hyperactivation (45). Similarly, *STAT1* activation may induce antiproliferative and proapoptotic responses and enhance antitumor immunity during carcinogenesis, while *STAT3* activation enhances tumor cell survival/proliferation, motility, and immune tolerance (46). As seen in **Supplementary Figure 9**, among the DEGs between ATB and LTBI, regulation by *STAT3* is more significant than regulation by *STAT1*, implying that patients with ATB may have more compensatory activation of *STAT3* than those with LTBI.

To obtain comprehensive and complementary benefits, we applied DEG analysis, GSEA, WGCNA, and other analytic methodologies to analyze transcriptional profiling in our study. It is obvious that, regardless of the method, interferon signaling pathways (including interferon alpha/beta, interferon gamma) and responses to the virus can be significantly enriched. Type I and type II interferons are important regulators of immunity and inflammation, which activate the Jak–STAT signaling pathway and induce the expression of interferon-stimulated genes encoding antiviral responses, inflammation, antigen presentation, and autoimmunity (47). About 10% genes in the human genome are potentially regulated by interferons. We observed high expression of many interferon-stimulated genes in the ATB group, including guanylate-binding proteins (*GBP1, GBP2, GBP3, GBP4, GBP5*); interferon-inducible proteins (*IFI6, IFIH1, IFIT2, IFIT3, IFITM1, IFITM3*); and *OAS1; OAS3; RSAD2; STAT1; STAT2;* and *TRIM22*. GBPs mediate a broad spectrum of innate immune functions against a wide variety of microbial pathogens (48). It can be seen that after *M. tuberculosis* infection, neutrophils are also actively involved in the production of interferons to fight the infection. Although IFN-γ has a protective role in the immune response to intracellular pathogens, including mycobacteria, increased and sustained levels of type I interferons may contribute to the progression of tuberculosis (49).

We compared our differentially expressed gene list with previously published lists of tuberculosis and other disease-related genes, including influenza, chronic obstructive pulmonary disease (COPD), community-acquired pneumonia (CAP), and sarcoidosis (**Supplementary Table 6**). Many interferon pathway-related genes, including STAT1,GBP1,GBP5, IFI44, IFIH1, FITM3, have been described in whole-blood transcriptome studies of tuberculosis. In influenza patients, the interferon pathway-related genes IFI44, IFI6, IFIH1, STAT1, and GBP1 also showed overexpression, while no obvious interferon transcriptional signature was observed in AECOPD and CAP. Notably, we found that the ATF3 gene, which is involved in the complex process of cellular stress response, has expression changes in tuberculosis, influenza, AECOPD and CAP.

In addition, our study also found some undetected differences in the whole blood studies, such as genes related to neutrophil migration chemotaxis, CXCR4, CXCL8 and ICAM1, which were significantly highly expressed in ATB. Chemokines CXCR4 and CXCL12 can regulate the retention and release of neutrophils in bone marrow and the homing and clearance of aging neutrophils. In mice, increased expression of CXCR4 contributes to the rapid migration of neutrophils to sites of inflammation (50). Similarly, high expression of CXCL8 also promotes the recruitment of neutrophils to the site of infection.

The neutrophil gene expression pattern of LTBI is similar to that of HC, but we discovered that LTBI was downregulated in the RNA splicing pathway in comparison to HCs, although the exact mechanism is yet unknown. However, it is widely accepted that host cell alternative splicing plays a critical role in pathogen infection and immune regulation, as well as in the inflammatory processes (51), and it has been reported that infection of macrophages with *M. tuberculosis* results in the production of large amounts of macrophage RNA alternative splicing, which impairs normal protein expression (52).

Despite the presence of *M. tuberculosis* infection in both, the gene expression patterns of neutrophils in LTBI and ATB were significantly different. This also reflects the difference in the functional activation state of neutrophils in these two infection states. That is, in active tuberculosis, neutrophils function are active and in LTBI, they are in quiescent states. Previously, it was reported that the gene expression pattern of TB-like patients in IGRA-positive LTBI were closer to TB (53, 54). Tabone’s research shows that there was an increasing differential expression in published signatures as the disease progresses, from the incipient TB to subclinical TB, and clinical TB (15). The LTBI patients included in this study were all IGRA-positive, and subclinical infections with pulmonary lesions were excluded by chest X-ray, so we didn’t observe significant heterogeneity within LTBIs.

Bacteriological confirmation is the gold standard for TB diagnosis, but the diagnosis rate is poor and has not improved for many years. The study of host transcriptomics can reflect the state of host immune response, which is not only conducive to understanding the occurrence and development of tuberculosis, but also has great potential to improve clinical diagnosis of tuberculosis and indicate the progression from latent infection to active disease. There have also been many previous studies of blood transcriptional biomarkers for active pulmonary tuberculosis, mostly based on whole blood or PBMC samples (55). In this study, we validated the ability of four genes screened by high-throughput sequencing to discriminate ATB in neutrophil subsets by qPCR. It provides clues for the study of transcriptional markers for auxiliary diagnosis of tuberculosis in the future.

This study has certain limitations. The relatively small sample size of our ATB group was related to our stringent sample inclusion criteria, such as newly diagnosed and initial treated patients with bacteriologically confirmed disease. Although the patients in the ATB group were all newly diagnosed, the duration of their infection was uncertain and the severity of disease varied. We used the CD15 immunomagnetic positive sorting method for neutrophil sorting. Although the CD15+ cell population also contains a small number of other cells such as eosinophils, the absolute dominance of the proportion of neutrophils makes the gene expression profile of this type of cells representative of the gene expression profile of neutrophils. In addition, the results of transcriptome analysis only represent changes at the level of gene expression, as for the specific changes in protein levels and functional pathways, more in-depth mechanistic studies are needed.

In conclusion, utilizing high-throughput sequencing techniques and comprehensive analysis, our work illustrates the gene expression profile of peripheral blood neutrophils in the native state of hosts with different *M. tuberculosis* infection status. As the most abundant innate immune cell, neutrophils, during active tuberculosis infection, recognize pathogen-associated molecular patterns through a series of pattern recognition receptors and trigger downstream cellular events for host defense, especially interferon-induced associated pathways. However, the immune escape mechanisms of *M. tuberculosis* may interfere with neutrophil antibacterial functions, such as inhibition of phagocytosis, granule maturation, and neutrophil degranulation. In addition, *M. tuberculosis* may attenuate inflammation and apoptosis by inhibiting the NF-κB signaling pathway and promote its survival in neutrophils. By qPCR analysis, three novel transcriptional markers—*RBM3, CSRNP1*, and *SRSF5*—with the ability to discriminate ATB from LTBI and HC were identified.

## Data availability statement

The data presented in the study are deposited in the National Genomics Data Center (NGDC), accession number PRJCA010442.

## Ethics statement

The studies involving human participants were reviewed and approved by The Ethics Committees of the Institute of Pathogen Biology, Chinese Academy of Medical Sciences. The patients/participants provided their written informed consent to participate in this study.

## Authors contributions

QJ and XZ conceived and designed the study and provided guidance on manuscript writing. XG performed the lab work, statistical analysis and manuscript writing. XZ, XW, LL, and SL performed the lab work and statistical analysis. LG, QY, HX, BZ, DW, QC, ZL, MZ, and SP diagnosed and collected samples. All authors contributed to the manuscript revision and approved the submitted version.

## Funding

The present study was supported by CAMS Initiative for Innovative Medicine (2021-I2M-1-037, 2016-I2M-1-013), Jin Qi team of Sanmin Project of Medicine in Shenzhen, Sanmin Project of Medicine in Shenzhen (GCZX2015043015340574) and the prevention and treatment of AIDS, viral hepatitis and other infectious diseases” (2017ZX10201301-002-002), and the Non-profit Central Research Institute Fund of Chinese Academy of Medical Sciences (2020-PT310-004, 2021-PT310-004).

## Acknowledgments

We would like to thank the patients and other participants for cooperating with our study. We also thank the healthcare workers involved in patients’ care and samples collection.

## Conflict of interest

The authors declare that the research was conducted in the absence of any commercial or financial relationships that could be construed as a potential conflict of interest.

## Publisher’s note

All claims expressed in this article are solely those of the authors and do not necessarily represent those of their affiliated organizations, or those of the publisher, the editors and the reviewers. Any product that may be evaluated in this article, or claim that may be made by its manufacturer, is not guaranteed or endorsed by the publisher.
