# Supplementary material for: Whole transcriptome sequencing reveals neutrophils’ transcriptional landscape associated with active tuberculosis
Source: Front Immunol. 2022 Aug 18;13:954221. doi: 10.3389/fimmu.2022.954221 (PMC9436479; doi:10.3389/fimmu.2022.954221)
Supplement: Supplementary file 1 [file DataSheet_1.docx]

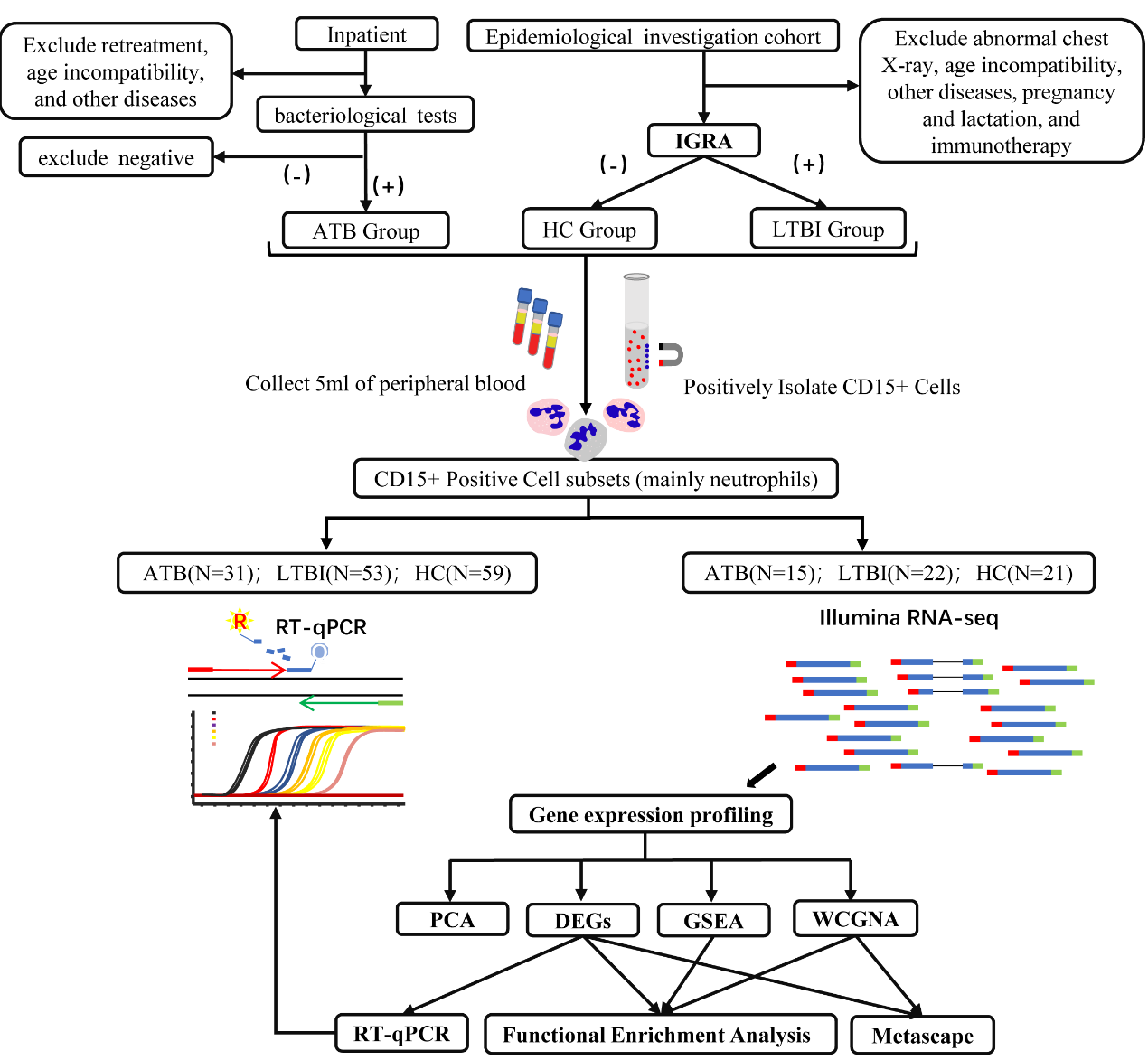
 **Supplementary Figure 1.** Schematic diagram of the overall study design and workflow.


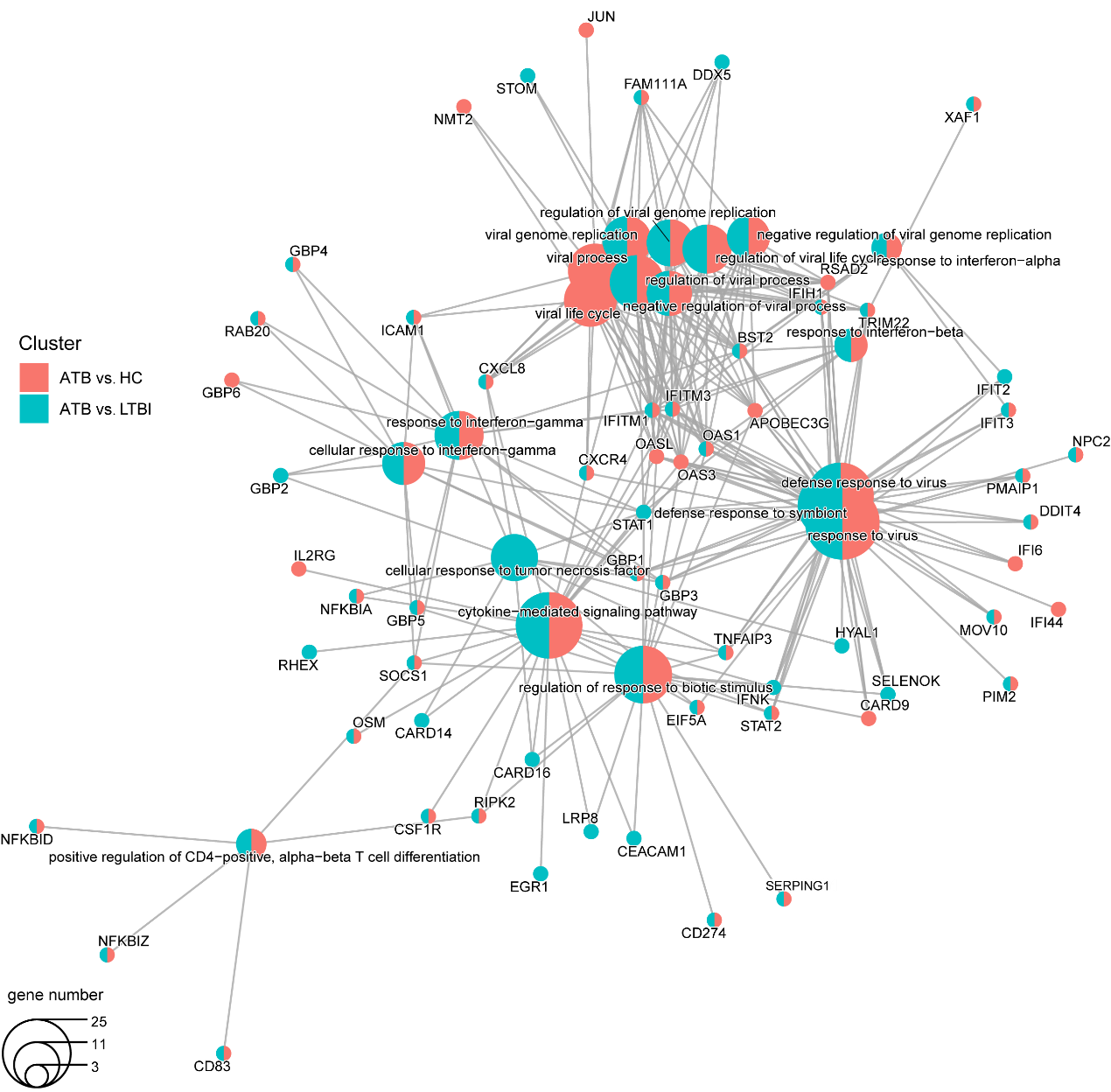


**Supplementary Figure 2.** Gene network plot of core enriched GO terms. Interferon-stimulated genes-related pathways, including defense response to virus, regulation of viral process, cytokine-mediated signaling pathway, and interferon-gamma signaling were significantly enriched. DEGs and enriched terms obtained from different comparison groups are distinguished by different colors.


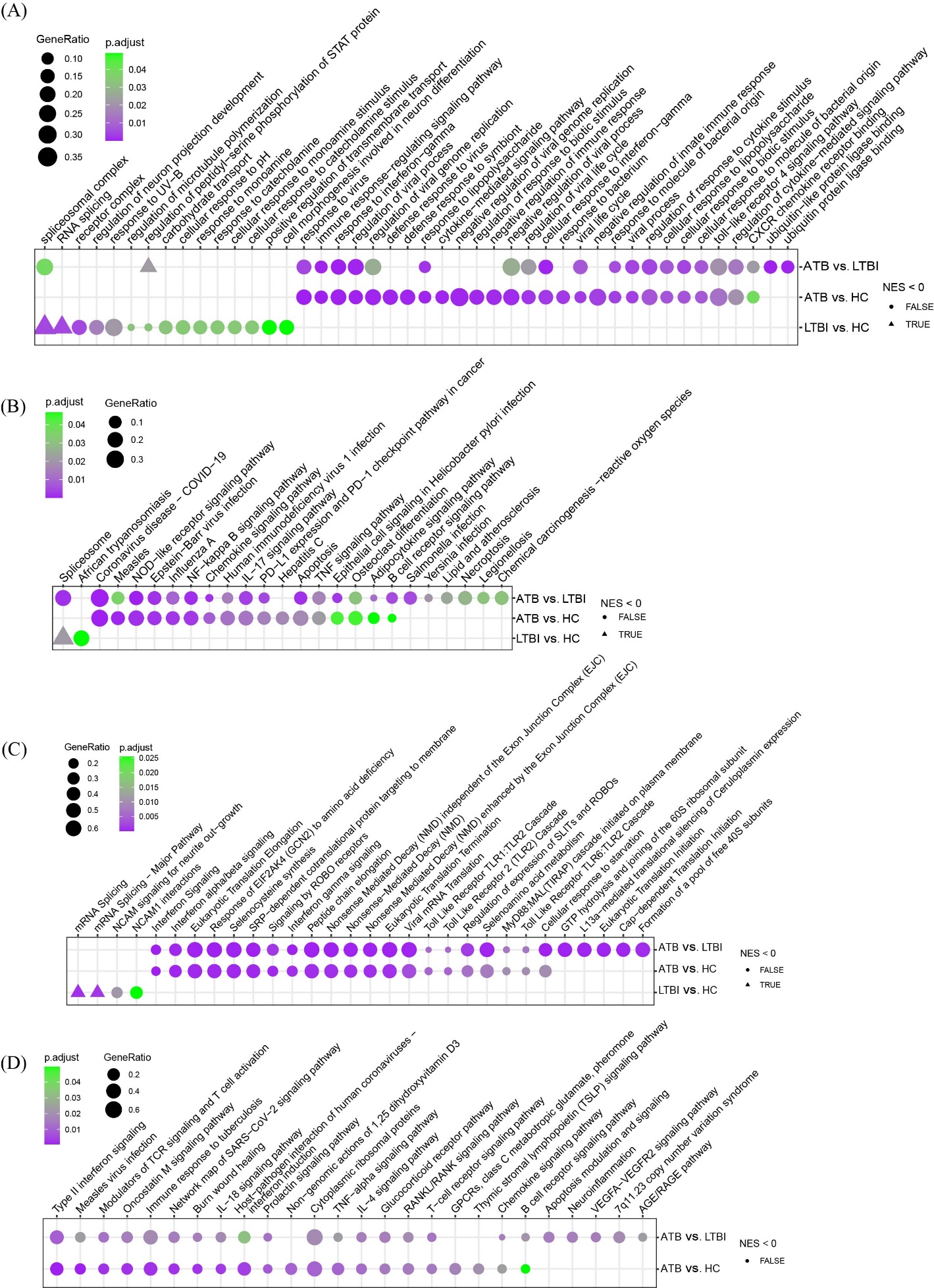


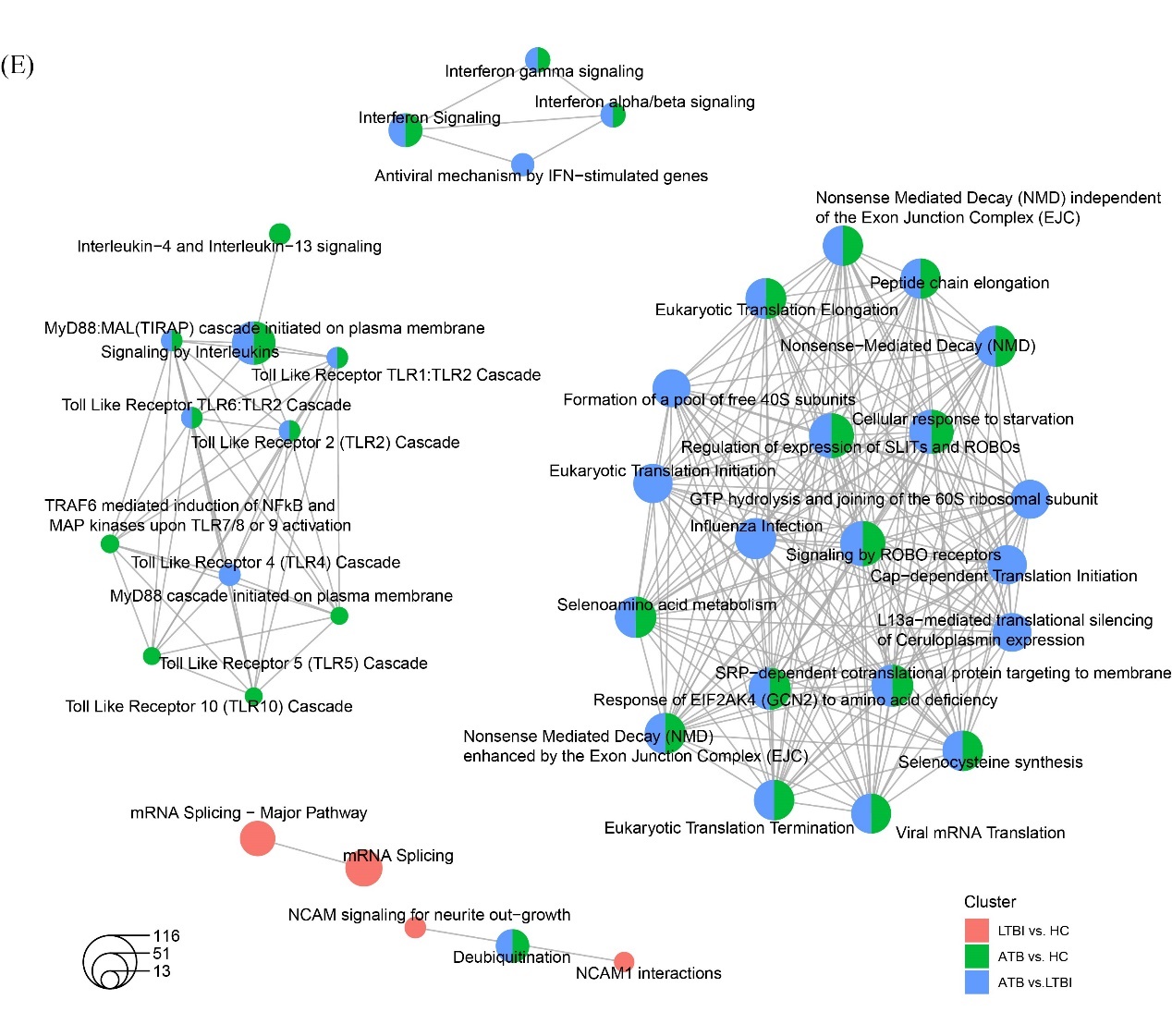
 **Supplementary Figure 3.** Gene set enrichment analysis bubble plot for pairwise comparisons. Genes are preranked in descending order of size by -log10 p value * log2 Foldchange. (A) GO Biological Process, (B) Reactome pathways, (C) WiKipathways, (D) KEGG pathways. The color of the bubbles indicates significance (adjusted P-value), and the size corresponds to the proportion of genes with corresponding annotation. Circles represent up-regulation, triangles represent down-regulation. (E) GSEA-Reactome network map with nodes representing the significant enriched reactome pathways. The colors of the nodes represent different comparison groups, the size of the nodes represents the number of genes enriched and each edge represents the overlap between two gene sets.


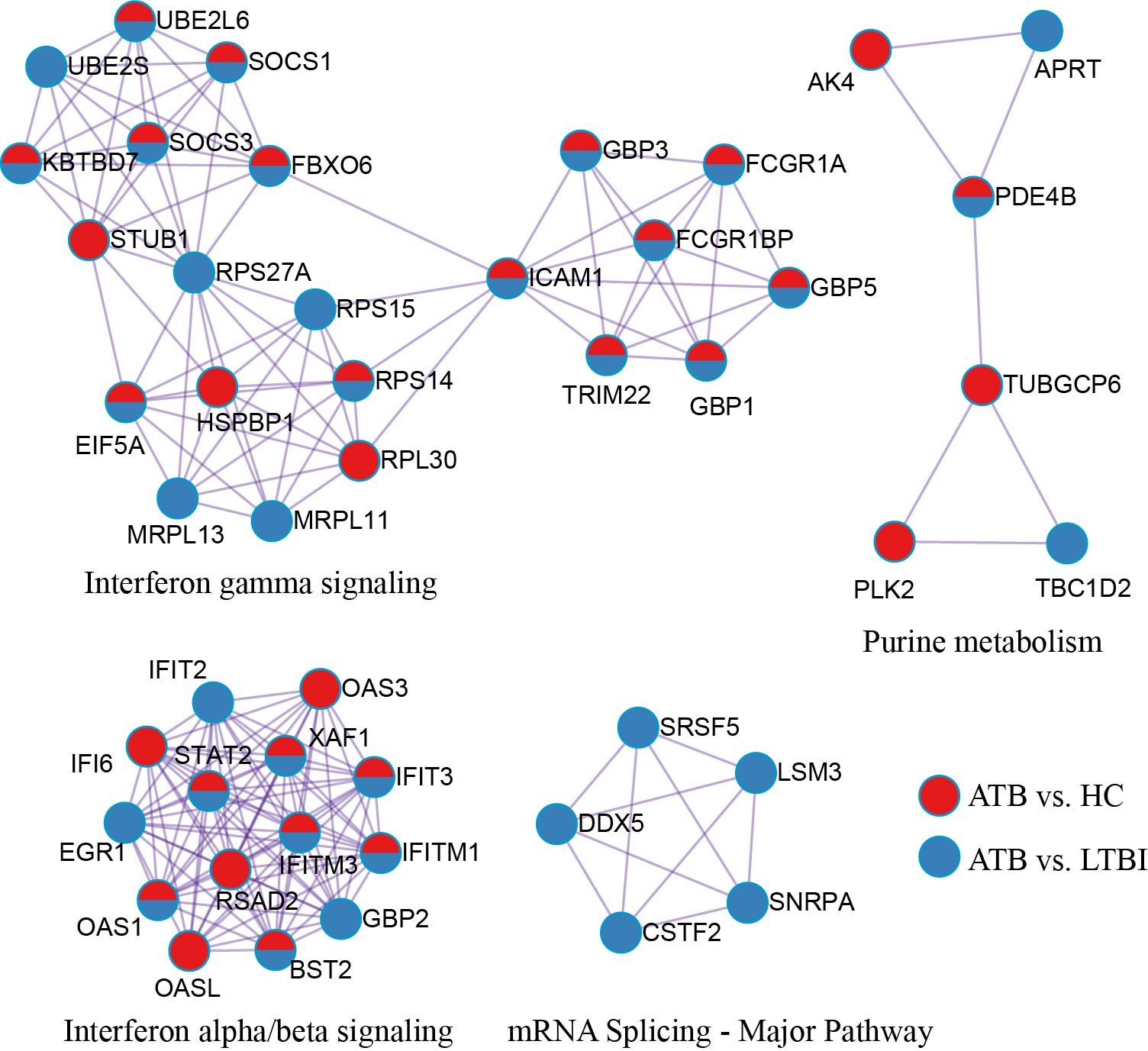


**Supplementary Figure 4.** The top 4 core PPI networks of DEGs and their significantly enriched functional pathways. Red dots represent DEGs in ATB vs. HC, and blue dots represent DEGs in ATB vs. LTBI.


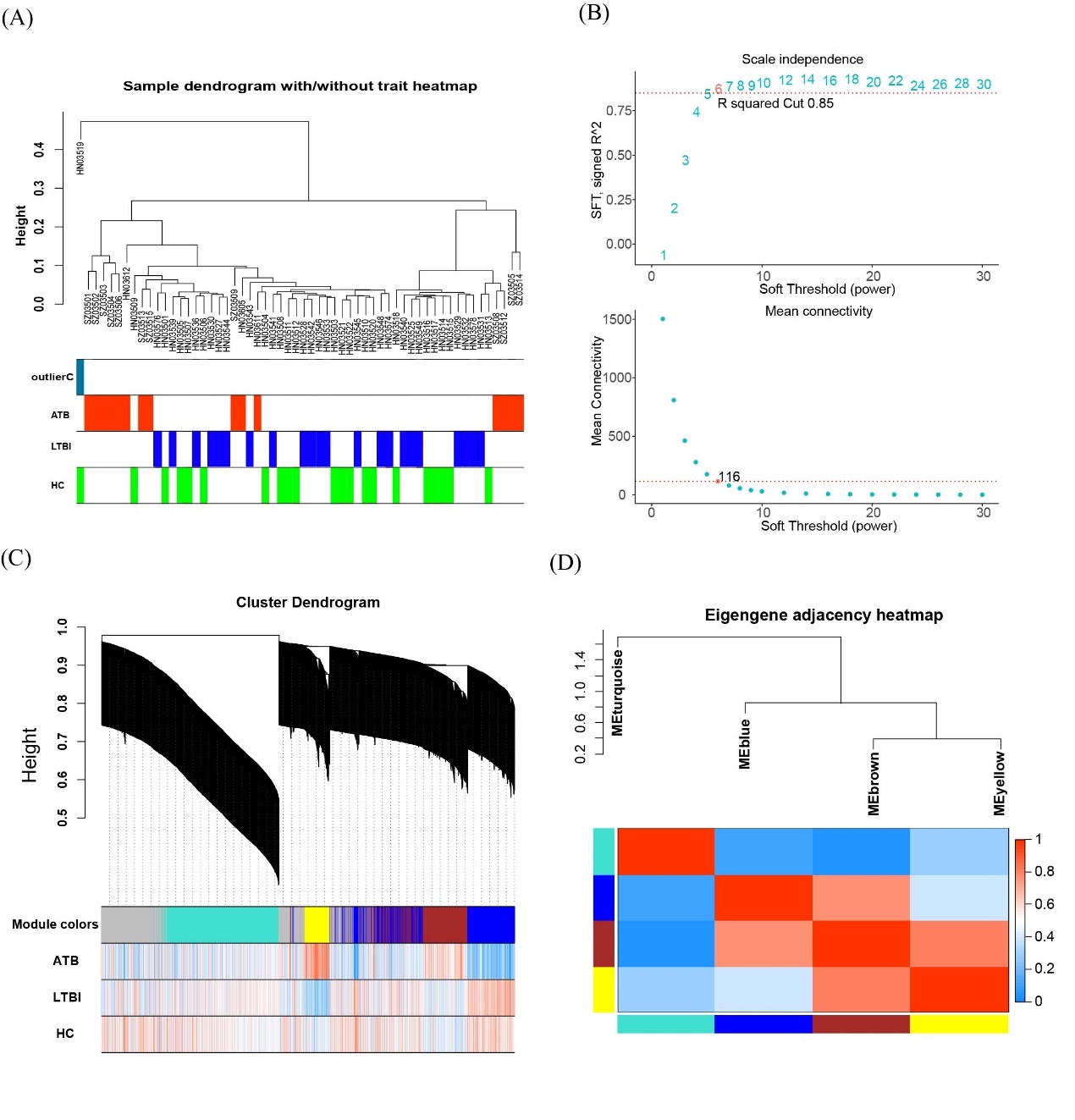


**Supplementary Figure 5.** Modular analysis using Weighted Gene Co‐expression Network Analysis (WGCNA). (A) Samples clustering tree and samples grouping distribution map. Hierarchical clustering analysis was applied to check the heterogeneity of all samples, and outlier sample was deleted to ensure the network's dependability was maintained. The upper part of the figure is the clustering tree of all samples, each row in the lower half represents a group of samples, the outlier row represents the outlier samples, and the bars with different colors correspond to the samples of the clustering tree above. (B) The selection of the optimal soft threshold, the figure shows the relationship between soft threshold and scale independence and mean connectivity. (C) Hierarchical clustering tree constructed from gene co-expression relationships. Each color in Module colors represents a co-expressed gene module, and genes that do not belong to any module are defined as gray. The bottom three rows represent the relative expression of each gene in different groups (red represents high expression and blue represents low expression). (D) Correlation of all identified modules.


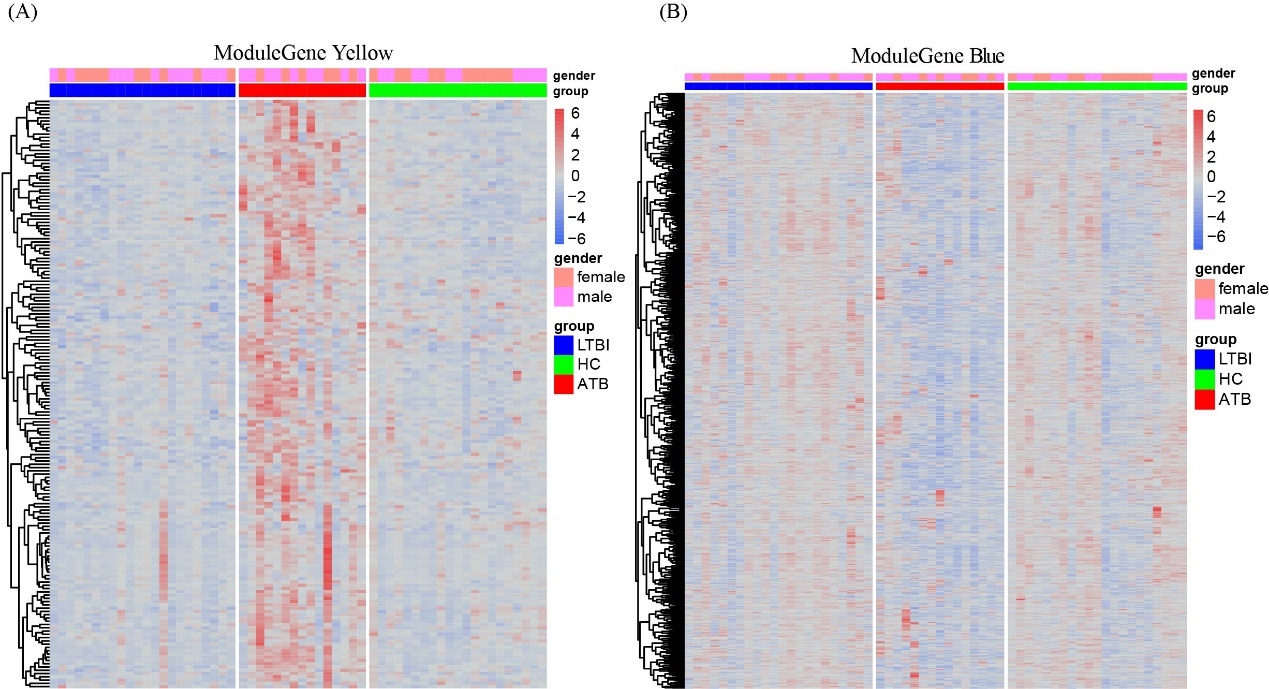


**Supplementary Figure 6** (A) and (B) Hierarchical clustering heatmap of blue module and yellow module genes. Each row represents a gene, columns represent samples, and relative levels of gene expression are represented by color scale, with red for high expression and blue for low expression.


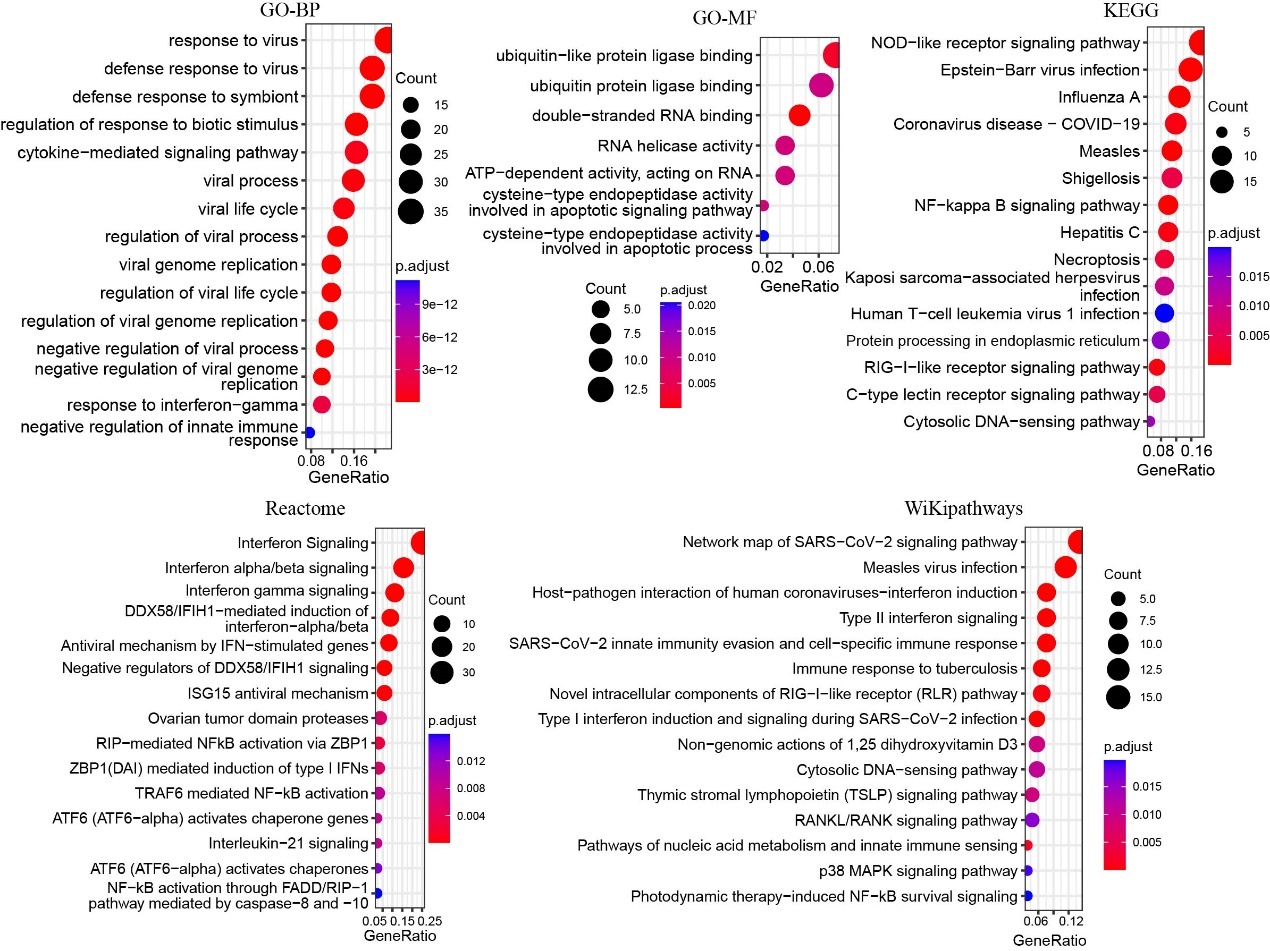


**Supplementary Figure 7.** Bubble plots of functional enrichment analysis of genes in yellow modules, showing only the top 15 significantly enriched pathways. The color of the bubbles indicates significance (adjusted P-value), and the size corresponds to the count of genes with corresponding annotation.


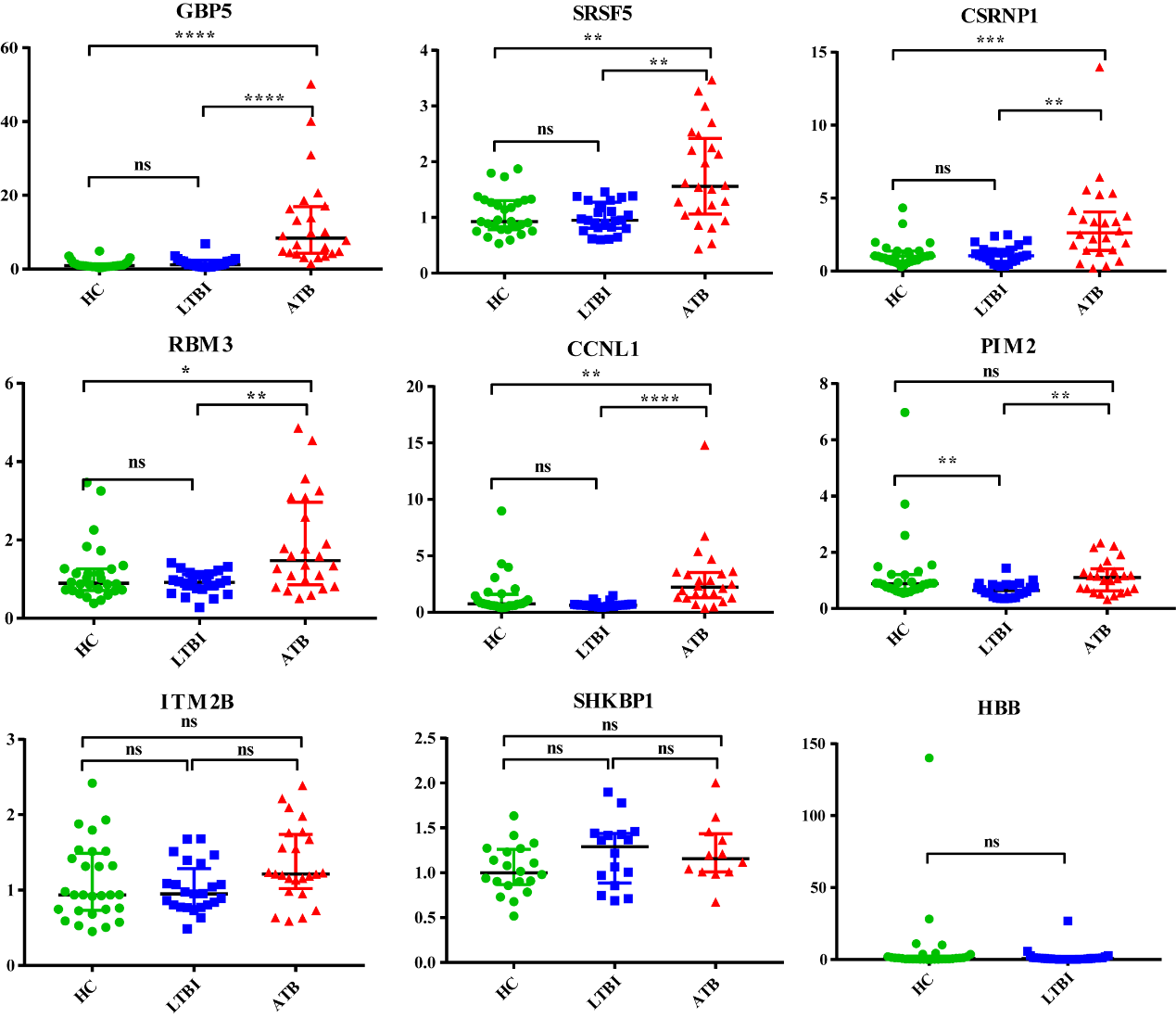
**Supplementary Figure 8** The expression of *GBP5, SRSF5, CSRNP1, RBM3,* and *CCNL1* genes were significantly increased in the ATB group. There was no statistical difference in the *PIM2* gene, but the overall dispersion was relatively high, and the trend was biased towards an increase. The negative control genes *SHKBP1* and *ITM2B* were not statistically different among the three groups. The only significant DEG *HBB* screened in LTBI vs. HC was verified to have no significant difference.


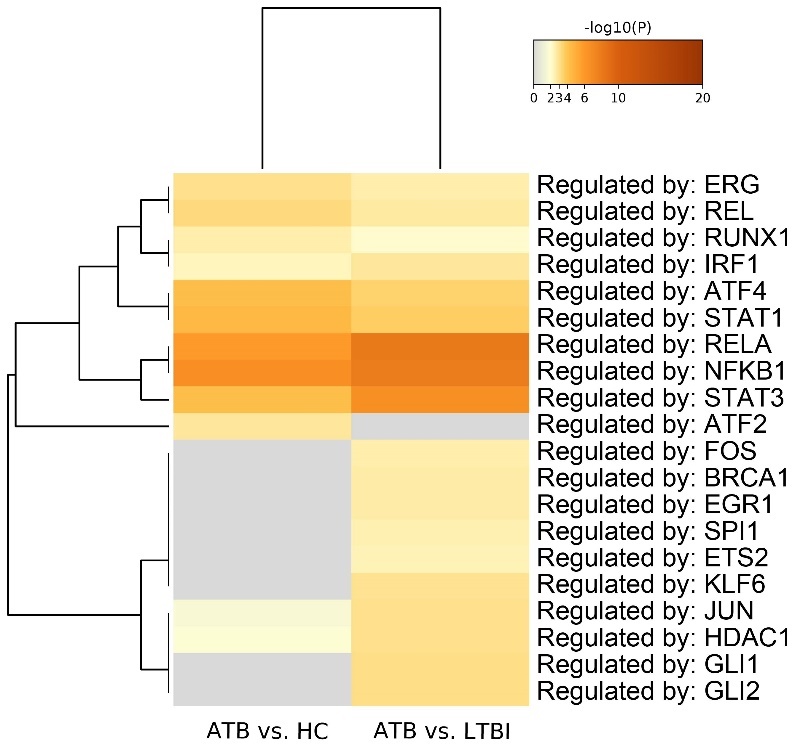


**Supplementary Figure 9.** Summary of enrichment analysis of differentially expressed genes in TRRUST. The analysis results showed that these differentially expressed genes were mainly regulated by *NFKB1, RELA, STAT3, STAT1* and *ATF4*.

**Supplementary Table 1. Samples information**

| Samples | Age | Gender | Bacteriological testing | Chest x-ray or CT | Others |
| --- | --- | --- | --- | --- | --- |
| 20SZ03501 | 37 | male | +++/+/+/+++ | cavities | chronic superficial gastritis, hepatic cyst |
| 20SZ03502 | 26 | male | +/+/+/++ | shadow | - |
| 20SZ03503 | 40 | female | ++/+/NA/+++ | shadow | endometrioma, ovarian cyst |
| 20SZ03504 | 50 | male | ++++/+/+/+++ | cavities, pleural thickening | type 2 diabetes, chronic hepatitis B, portal hypertension, Hypersplenism, right kidney stone, right hydronephrosis, malnutrition |
| 20SZ03505 | 20 | female | +++/+/NA/+++ | shadow, emphysema | - |
| 20SZ03506 | 25 | male | ++/+/+/++++ | cavities, shadow | - |
| 20SZ03508 | 30 | male | -/NA/+/++ | shadow, pleural thickening | - |
| 20SZ03509 | 52 | female | -/NA/NA/+ | nodular lesions, shadow | thrombocytopenic purpura |
| 20SZ03512 | 24 | female | NA/NA/NA/++ | NA | - |
| 20SZ03513 | 44 | male | +/NA/+/++++ | cavities | - |
| 20SZ03514 | 49 | female | +++/NA/NA/+++ | cavities, mediastinal lymphadenopathy | type 2 diabetes, chronic hepatitis B |
| 20SZ03515 | 38 | male | NA/NA/+/++ | pleural effusion | Paroxysmal Supraventricular Tachycardia |
| 20HN03605 | 56 | male | NA/NA/+/NA | shadow | NA |
| 20HN03611 | 56 | male | NA/NA/+/NA | shadow | NA |
| 20HN03612 | 58 | female | ++/NA/+/NA | shadow | NA |

Cohort for RNA-seq (ATB group)

Bacteriological testing: acid-fast staining smears/*M. tb* culture/*M. tb* -DNA PCR/Xpert MTB-RIF, +:positive, -: negative, NA: not available

Cohort for qPCR Validation (ATB group)

| Samples | Age | Gender | Bacteriological testing | Chest x-ray or CT | Others |
| --- | --- | --- | --- | --- | --- |
| 19SZ03002 | 30 | male | ++/+/+/+++ | cavities | - |
| 19SZ03003 | 18 | female | -/+/-/+ | pleural effusion, shadow | drug-induced liver damage |
| 19SZ03004 | 33 | male | -/NA/NA/+++ | shadow | - |
| 19SZ03005 | 51 | male | -/+/-/NA | pulmonary consolidation | chronic bronchitis, emphysema |
| 19SZ03007 | 23 | female | +/+/-/++ | NA | - |
| 19SZ03008 | 21 | male | +/+/-/++ | cavities, shadow | mitral regurgitation, arrhythmia |
| 19SZ03010 | 20 | male | -/+/-/- | shadow | - |
| 19SZ03011 | 25 | female | NA/NA/NA/+ | cavities, shadow | - |
| 19SZ03013 | 28 | female | -/+/-/+ | pleural effusion | - |
| 19SZ03015 | 40 | male | -/+/-/+++ | compensatory enlargement, multiple lesions | Chronic obstructive pulmonary disease |
| 19SZ03019 | 25 | male | +/+/+/+++ | cavities | Gallbladder Polyps |
| 19SZ03018 | 32 | male | -/+/+/++ | cavities, pleural effusion | - |
| 19SZ03021 | 51 | male | NA/+/NA/+ | shadow | - |
| 19SZ03026 | 22 | male | ++/+/NA/+++ | cavities | community acquired pneumonia, chronic urticaria |
| 19SZ03038 | 26 | female | NA/+/-/- | shadow | drug-induced liver damage |
| 19SZ03040 | 38 | male | -/+/NA/NA | nodular hyperdense shadow | fatty liver |
| 19SZ03041 | 48 | female | NA/+NA/+ | nodular calcification,pleural thickening | grade 2 hypertension |
| 19SZ03042 | 32 | female | -/+/-/++ | shadow | otitis media, sinusitis |
| 19SZ03044 | 29 | female | -/+/+/+ | pleural effusion | pericardial effusion |
| 19SZ03045 | 49 | male | ++++/+/+/+++ | shadow | - |
| 19SZ03047 | 69 | male | +++/NA/+/++++ | cavities | type 2 diabetes, Gallstone |
| 19SZ03046 | 25 | female | ++++/+/+/+++ | bronchial caseous necrosis | Mitral and tricuspid regurgitation, arrhythmia |
|  |  |  |  |  |  |
|  |  |  |  |  |  |
| 19SZ03050 | 57 | female | ++++/+/+/+++ | shadow | chronic hepatitis B |
| 19SZ03054 | 33 | male | +/+/+/+++ | cavities, pleural effusion | - |
| 19SZ03055 | 56 | male | -/+/-/- | cavities, emphysema | Chronic obstructive pulmonary disease, emphysema |
| 19SZ03056 | 25 | male | ++/+/+/+++ | cavities, shadow | Hyperuricemia |
| 19SZ03110 | 44 | female | -/+/-/+++ | shadow | chronic hepatitis B |
| 19SZ03111 | 23 | male | -/-/-/+++ | cavities | - |
| 19SZ03113 | 32 | female | +/+/NA/+++ | shadow | - |
| 19SZ03114 | 26 | female | -/+/+/+++ | nodular lesions, shadow | - |
| 19SZ03117 | 22 | male | +/-/-/- | pleural thickening, shadow | tuberculous meningitis |

Bacteriological testing: acid-fast staining smears/*M. tb* culture/*M. tb* -DNA PCR/Xpert MTB-RIF, +:positive, -: negative, NA: not available

Characteristics of different groups of samples

| **Cohort for RNA-seq** | **Group** | **ATB** | **LTBI** | **HC** |
| --- | --- | --- | --- | --- |
|  | Numbers | 15 | 22 | 21 |
|  | Gender(Male/Female) | 9/6 | 12/10 | 10/11 |
|  | Age(average) | 20-58（40） | 53-60（56） | 55-60（57） |
|  | Clinical symptoms | Symptomatic^1^ | Asymptomatic^2^ | Asymptomatic^2^ |
|  | IGRA | NA | Positive | Positive |
|  | Bacteriological testing | Positive^3^ | NA | NA |
|  | Chest x-ray or CT | Abnormal^4^ | Normal^5^ | Normal^5^ |
| **Cohort for qPCR Validation** | Numbers | 31 | 53 | 59 |
|  | Gender(Male/Female) | 18/13 | 28/25 | 28/31 |
|  | Age(average) | 22-57（41） | 54-60（57） | 53-60（57） |
|  | Clinical symptoms | Symptomatic^1^ | Asymptomatic^2^ | Asymptomatic^2^ |
|  | IGRA | NA | Positive | Positive |
|  | Bacteriological testing | Positive^3^ | NA | NA |
|  | Chest x-ray or CT | Abnormal^4^ | Normal^5^ | Normal^5^ |

1: All ATB patients were hospitalized patients with different degrees of respiratory symptoms, such as cough, blood-stained sputum, hemoptysis, etc.; 2: All LTBI and HC participants excluded those with tuberculosis- and respiratory-related clinical phenotypes; 3: The test items and results of each sample are shown in the above detailed information table; 4: The chest x-ray or CT appearance of each sample is shown in the above detailed information table; 5: All LTBI and HC participants excluded patients with abnormal chest x-ray; NA: No relevant test data.

**Supplementary Table 2. RNA-seq Data Summary**

| Groups | Samples | Raw Base(G) | Clean Base(G) | Q30(%) |
| --- | --- | --- | --- | --- |
| HC | 20HN03501 | 4.09 | 2.85 | 92.84 |
| HC | 20HN03502 | 13.59 | 9.14 | 92.27 |
| HC | 20HN03503 | 6.16 | 4.35 | 92.77 |
| HC | 20HN03504 | 5.82 | 4.6 | 92.44 |
| HC | 20HN03505 | 4.04 | 3.19 | 92.85 |
| HC | 20HN03506 | 4.99 | 2.94 | 93.25 |
| HC | 20HN03507 | 4.59 | 3.06 | 92.94 |
| HC | 20HN03508 | 5.41 | 3.49 | 92.56 |
| HC | 20HN03509 | 4.67 | 3.13 | 92.77 |
| HC | 20HN03510 | 6.92 | 4.65 | 92.94 |
| HC | 20HN03511 | 5.85 | 3.9 | 92.59 |
| HC | 20HN03512 | 6.28 | 4.56 | 93.21 |
| HC | 20HN03513 | 3.69 | 2.38 | 92.67 |
| HC | 20HN03514 | 5.57 | 3.87 | 92.88 |
| HC | 20HN03515 | 5.98 | 4.38 | 93.1 |
| HC | 20HN03516 | 5.64 | 3.69 | 93.45 |
| HC | 20HN03517 | 4 | 2.58 | 93.07 |
| HC | 20HN03518 | 5.32 | 2.55 | 93.16 |
| HC | 20HN03519 | 5.27 | 3.71 | 92.48 |
| HC | 20HN03520 | 4.82 | 3.37 | 92.9 |
| HC | 20HN03521 | 4.85 | 4.18 | 92.98 |
| LTBI | 20HN03522 | 6.02 | 4.49 | 92.9 |
| LTBI | 20HN03525 | 5.07 | 3.12 | 93.02 |
| LTBI | 20HN03527 | 7.13 | 4.3 | 93.17 |
| LTBI | 20HN03528 | 7.49 | 5.49 | 93.28 |
| LTBI | 20HN03529 | 5.82 | 3.97 | 93.22 |
| LTBI | 20HN03530 | 6.39 | 4.39 | 93.53 |
| LTBI | 20HN03531 | 5.53 | 2.98 | 92.92 |
| LTBI | 20HN03532 | 11.2 | 7.7 | 93.16 |
| LTBI | 20HN03533 | 3.79 | 2.64 | 92.23 |
| LTBI | 20HN03536 | 4.52 | 3.32 | 93.42 |
| LTBI | 20HN03540 | 4.91 | 3.1 | 92.49 |
| LTBI | 20HN03541 | 4.02 | 2.64 | 92.32 |
| LTBI | 20HN03542 | 5.56 | 3.78 | 92.42 |
| LTBI | 20HN03543 | 6.78 | 4.56 | 92.42 |
| LTBI | 20HN03544 | 5.79 | 3.85 | 92.98 |
| LTBI | 20HN03545 | 4.61 | 3.1 | 92.43 |
| LTBI | 20HN03546 | 6.38 | 3.85 | 91.9 |
| LTBI | 20HN03548 | 5.72 | 3.41 | 92.51 |
| LTBI | 20HN03549 | 5.09 | 3.48 | 93.08 |
| LTBI | 20HN03574 | 6.16 | 3.33 | 93.23 |
| LTBI | 20HN03576 | 4.51 | 2.32 | 93.46 |
| LTBI | 20HN03578 | 4.34 | 2.94 | 93.02 |
| ATB | 20HN03605 | 4.64 | 2.01 | 92.76 |
| ATB | 20HN03611 | 5.74 | 3.23 | 92.75 |
| ATB | 20HN03612 | 5.81 | 3.05 | 93.18 |
| ATB | 20SZ03501 | 6.06 | 2.37 | 93.94 |
| ATB | 20SZ03502 | 5.33 | 4.01 | 95.27 |
| ATB | 20SZ03503 | 5.44 | 3.27 | 95.12 |
| ATB | 20SZ03504 | 5.52 | 3.25 | 95.19 |
| ATB | 20SZ03505 | 5.54 | 3.35 | 95.19 |
| ATB | 20SZ03506 | 5.37 | 3.58 | 95.44 |
| ATB | 20SZ03508 | 10.3 | 7.1 | 94.7 |
| ATB | 20SZ03509 | 4.95 | 3.86 | 93.91 |
| ATB | 20SZ03512 | 6.2 | 5.21 | 95.19 |
| ATB | 20SZ03513 | 5.25 | 4.66 | 94.6 |
| ATB | 20SZ03514 | 5.69 | 4.99 | 94.44 |
| ATB | 20SZ03515 | 4.5 | 4.19 | 94.55 |

**Supplementary Table 3. qPCR-TaqMan™ Gene Expression Assay**

| Gene Symbol | Assay ID | Amplicon length | Assay Design |
| --- | --- | --- | --- |
| CSRNP1 | Hs01042626_g1 | 148 | Probe spans exons |
| PIM2 | Hs00179139_m1 | 84 | Probe spans exons |
| CCNL1 | Hs01089784_m1 | 92 | Probe spans exons |
| SRSF5 | Hs00951036_g1 | 153 | Probe spans exons |
| RBM3 | Hs00943160_g1 | 72 | Probe spans exons |
| GBP5 | Hs00369472_m1 | 73 | Probe spans exons |
| ITM2B | Hs00222753_m1 | 77 | Probe spans exons |
| SHKBP1 | Hs00370710_m1 | 60 | Probe spans exons |
| HBB | Hs00758889_s1 | 95 | Probe spans exons |
| MYO1F | Hs01027595_m1 | 60 | Probe spans exons |

We selected nine target genes and used qPCR to verify the accuracy of RNA-seq results. Among them, *GBP5* has been reported to have a significant correlation with active tuberculosis, and is relatively highly expressed in ATB patients, and the other genes have not been reported to be related to tuberculosis. The other five genes (*CSRNP1, PIM2, CCNL1, SRSF5, RBM3*) were selected based on factors such as fold change, significance, and gene functions. *SHKBP1* and *ITM2B* showed no significant difference among the groups as negative controls, and HBB was the only differentially expressed gene screened in LTBI vs. HC.

| **Supplementary table 6. Comparison With Published Gene Lists** | | | | | | | | |
| --- | --- | --- | --- | --- | --- | --- | --- | --- |
| Study | This study | | Andres-Terre et al ^[1]^ | Bertrams et al ^[2]^ | Bertrams et al ^[2]^ | Bloom et al ^[3]^ | Berry et al ^[4]^ | Berry et al ^[4]^ |
| Sample type | Neutrophils | | Whole blood | PBMCs | PBMCs | Whole blood | Whole blood | Whole blood |
| Gene lists | ATB vs. HC | ATB vs. LTBI | influenza infection | AECOPD vs. Healthy | CAP vs. Healthy | TB vs. active sarcoidosis | 393 genes | 86 genes |
| ACOT9 | - | ACOT9 | - | - | - | - | ACOT9 | - |
| ADM | ADM | ADM | - | - | - | ADM | ADM | - |
| ANKRD22 | - | ANKRD22 | - | - | - | - | ANKRD22 | - |
| APOL1 | APOL1 | APOL1 | - | - | - | - | APOL1 | - |
| APOL6 | - | APOL6 | APOL6 | - | - | - | APOL6 | - |
| ATF3 | ATF3 | ATF3 | ATF3 | ATF3 | ATF3 | - | ATF3 | - |
| BATF2 | BATF2 | BATF2 | - | - | - | - | BATF2 | - |
| BLVRB | - | BLVRB | - | BLVRB | BLVRB | - | - | - |
| BST2 | BST2 | BST2 | BST2 | - | - | - | - | - |
| CASP5 | CASP5 | CASP5 | - | - | - | - | CASP5 | - |
| CD274 | CD274 | CD274 | - | - | - | - | CD274 | - |
| CEACAM1 | - | CEACAM1 | - | - | - | CEACAM1 | CEACAM1 | - |
| CMPK2 | CMPK2 | - | - | - | - | - | CMPK2 | - |
| DUSP5 | DUSP5 | DUSP5 | - | - | - | - | - | - |
| EPSTI1 | EPSTI1 | EPSTI1 | - | - | - | - | EPSTI1 | - |
| ETV7 | - | ETV7 | - | - | - | - | ETV7 | - |
| FBXO6 | FBXO6 | FBXO6 | - | - | - | - | FBXO6 | - |
| FCGR1A | FCGR1A | FCGR1A | - | - | - | FCGR1A | FCGR1A | - |
| FCGR1B | FCGR1B | FCGR1B | - | - | - | FCGR1B | FCGR1B | - |
| FKBP5 | FKBP5 | - | - | - | - | FKBP5 | - | - |
| GADD45B | GADD45B | GADD45B | GADD45B | - | - | - | GADD45B | - |
| GBP1 | GBP1 | GBP1 | GBP1 | - | - | - | GBP1 | - |
| GBP2 | - | GBP2 | - | - | - | - | GBP2 | - |
| GBP4 | GBP4 | GBP4 | - | - | - | - | GBP4 | - |
| GBP5 | GBP5 | GBP5 | - | - | - | - | GBP5 | - |
| GBP6 | GBP6 | - | - | - | - | - | GBP6 | - |
| GSTK1 | GSTK1 | - | - | - | - | - | - | GSTK1 |
| HMGCR | - | HMGCR | - | HMGCR | - | - | - | - |
| HNRNPDL | - | HNRNPDL | - | HNRNPDL | - | - | - | - |
| IER5 | IER5 | IER5 | - | - | IER5 | - | - | - |
| IFI44 | IFI44 | - | IFI44 | - | - | - | IFI44 | - |
| IFI6 | IFI6 | - | IFI6 | - | - | - | IFI6 | - |
| IFIH1 | IFIH1 | IFIH1 | IFIH1 | - | - | - | IFIH1 | - |
| IFIT2 | - | IFIT2 | IFIT2 | - | - | - | IFIT2 | - |
| IFIT3 | IFIT3 | IFIT3 | IFIT3 | - | - | - | IFIT3 | - |
| IFITM1 | IFITM1 | IFITM1 | - | - | - | IFITM1 | IFITM1 | - |
| IFITM3 | IFITM3 | IFITM3 | - | - | - | IFITM3 | IFITM3 | - |
| IKZF5 | IKZF5 | - | - | - | IKZF5 | - | - | - |
| JMJD6 | - | JMJD6 | - | - | - | JMJD6 | - | - |
| LACTB | - | LACTB | - | - | - | - | LACTB | LACTB |
| LAP3 | - | LAP3 | LAP3 | - | - | - | LAP3 | - |
| MARC1 | - | MARC1 | - | MARC1 | - | - | - | - |
| NAPA | NAPA | NAPA | NAPA | - | - | - | - | - |
| NMT2 | NMT2 | - | - | NMT2 | - | - | - | - |
| NPC2 | NPC2 | NPC2 | - | - | - | - | - | NPC2 |
| OAS1 | OAS1 | OAS1 | OAS1 | - | - | - | OAS1 | - |
| OAS3 | OAS3 | - | OAS3 | - | - | - | OAS3 | - |
| OASL | OASL | - | OASL | - | - | - | OASL | - |
| ORM1 | ORM1 | ORM1 | - | ORM1 | ORM1 | - | - | - |
| OSM | OSM | OSM | - | - | - | OSM | OSM | - |
| P2RY14 | P2RY14 | P2RY14 | - | - | - | - | P2RY14 | - |
| PDE4B | PDE4B | PDE4B | - | - | PDE4B | - | - | - |
| PIM3 | - | PIM3 | - | - | - | PIM3 | - | - |
| PLAG1 | PLAG1 | PLAG1 | - | PLAG1 | PLAG1 | - | - | - |
| PLAUR | - | PLAUR | - | - | - | - | PLAUR | - |
| POLB | POLB | - | - | - | - | - | - | POLB |
| PSTPIP2 | PSTPIP2 | PSTPIP2 | - | - | - | - | PSTPIP2 | - |
| RAB20 | RAB20 | RAB20 | - | - | - | - | RAB20 | - |
| RALGAPA1 | - | RALGAPA1 | - | RALGAPA1 | - | - | - | - |
| RBM3 | RBM3 | - | - | RBM3 | - | - | - | - |
| RGS1 | - | RGS1 | - | RGS1 | RGS1 | - | - | - |
| RSAD2 | RSAD2 | - | RSAD2 | - | - | - | RSAD2 | - |
| SEPT4 | SEPT4 | - | SEPT4 | - | - | - | - | - |
| SERPING1 | SERPING1 | SERPING1 | SERPING1 | - | - | - | SERPING1 | - |
| SOCS1 | SOCS1 | SOCS1 | - | - | - | - | SOCS1 | - |
| SOCS3 | SOCS3 | SOCS3 | - | - | - | - | SOCS3 | - |
| SOWAHD | SOWAHD | - | - | SOWAHD | - | - | - | - |
| STAT1 | - | STAT1 | STAT1 | - | - | - | STAT1 | - |
| STAT2 | STAT2 | STAT2 | - | - | - | - | STAT2 | - |
| STMN3 | STMN3 | STMN3 | - | STMN3 | - | - | - | - |
| STOM | - | STOM | - | - | - | - | STOM | - |
| TRIM22 | TRIM22 | TRIM22 | - | - | - | - | TRIM22 | - |
| TSPYL5 | - | TSPYL5 | - | TSPYL5 | - | - | - | - |
| UBE2L6 | UBE2L6 | UBE2L6 | - | - | - | - | UBE2L6 | - |
| XAF1 | XAF1 | XAF1 | XAF1 | - | - | - | XAF1 | - |
| ZBTB10 | ZBTB10 | - | - | ZBTB10 | - | - | - | - |

**REFERENCES**

1. Andres-Terre M, McGuire HM, Pouliot Y, Bongen E, Sweeney TE, Tato CM, Khatri P. Integrated, Multi-cohort Analysis Identifies Conserved Transcriptional Signatures across Multiple Respiratory Viruses. Immunity. 2015 Dec 15;43(6):1199-211.

2. Bertrams W, Griss K, Han M, Seidel K, Klemmer A, Sittka-Stark A, et al. Transcriptional analysis identifies potential biomarkers and molecular regulators in pneumonia and COPD exacerbation. Sci Rep. 2020 Jan 14;10(1):241.

3. Bloom CI, Graham CM, Berry MP, Rozakeas F, Redford PS, Wang Y, Xu Z, et al. Transcriptional blood signatures distinguish pulmonary tuberculosis, pulmonary sarcoidosis, pneumonias and lung cancers. PLoS One. 2013 Aug 5;8(8):e70630. doi: 10.1371/journal.pone.0070630. Erratum in: PLoS One. 2013;8(8).

4. Berry MP, Graham CM, McNab FW, Xu Z, Bloch SA, Oni T, et al. An interferon-inducible neutrophil-driven blood transcriptional signature in human tuberculosis. Nature. 2010;466(7309):973-7.
